# Distributed Consensus Control for Connected Vehicle Platoon with Markovian Switching Topology and Heterogeneous Time-Varying Delays

**DOI:** 10.3390/s26206508

**Published:** 2026-10-14

**Authors:** Ruijia Li, Guokuan Yu, Peng Li, Di Ao, Xianbo Wang

**Affiliations:** 1 School of Mechanical and Electrical Engineering, Guizhou Normal University, Guiyang 550025, China; 18628117508@163.com (R.L.); lipeng@zjut.edu.cn (P.L.); 2 Institute of Facility Agriculture, Guangdong Academy of Agricultural Sciences, Guangzhou 510640, China; aodi@gdaas.cn; 3 Hainan Institute of Zhejiang University, Sanya 572025, China; xbwang@zju.edu.cn

**Keywords:** continuous-time Markov chain, cooperative control, time-varying delays, vehicle platoon

## Abstract

Reliable cooperative control of connected vehicle platoons remains challenging when stochastic communication-topology variations coexist with heterogeneous time-varying transmission delays. This study investigates a distributed consensus control problem for connected vehicle platoons subject to Markovian switching topologies and heterogeneous time-varying inter-vehicle communication delays. A third-order longitudinal vehicle model with powertrain inertial lag is combined with a continuous-time Markov chain (CTMC) to describe randomly switching communication topologies. A distributed control protocol incorporating the constant time headway policy, full velocity difference car-following behavior, and leader-pinning information is formulated, while link-dependent time-varying delays are embedded in the closed-loop error system. Based on a mode-dependent Lyapunov–Krasovskii functional, Jensen’s integral inequality, and the Schur complement, sufficient linear matrix inequality (LMI) conditions are derived for mean-square asymptotic stability of the nominal delayed system; the LMI is feasible at ${\bar{\tau }}_{\ell }=0.10$ s and $\mu_{\ell }=0.04$, remains feasible at 0.30 s, and becomes infeasible at 0.375 s. An input-to-state stability (ISS) corollary extends the result to bounded time-varying leader acceleration, showing that tracking errors remain bounded during dynamic EUDC phases and converge when the leader acceleration and its derivative vanish. Using the same Lyapunov–Krasovskii certificate, a mean-square $L_{\infty }$ bounded-propagation result is further established; this result characterizes uniform-in-vehicle error bounds and does not assert strict non-amplification. Numerical simulations under the Extra-Urban Driving Cycle (EUDC) show that the follower vehicles maintain cooperative tracking under topology switching and bounded communication delays, with position, velocity, and acceleration errors remaining bounded during the dynamic phases and converging as the leader acceleration vanishes. In the EUDC simulation, the largest leader-relative position error is approximately −60.75 m for Vehicle 10 at t≈380 s, the maximum adjacent local spacing-error peak ratio is 2.32, and the position error returns below 1 m by t≈400.8 s. The minimum inter-vehicle gap is 5.87 m and the minimum safety margin after the initial 10 s is 0.84, with all safety-margin indices remaining positive; these results indicate that the proposed framework maintains platoon coordination and car-following safety under stochastic communication uncertainties.

## 1. Introduction

Cooperative platoon control can effectively shorten inter-vehicle spacing, thereby improving road traffic efficiency, alleviating congestion, and enhancing driving safety. It has therefore emerged as a prominent research topic within intelligent transportation systems [1,2]. Unlike conventional adaptive cruise control (ACC), cooperative adaptive cruise control (CACC) leverages vehicle-to-everything (V2X) communication to enable following vehicles to acquire the motion states and control intentions of preceding vehicles in advance, thus improving the dynamic response of multi-vehicle systems in the presence of traffic-flow disturbances [3]. However, in real-world driving conditions, the performance of cooperative vehicle control is jointly influenced by heterogeneous vehicle dynamics, varying communication topologies, and information-transmission delays [4].

For multi-vehicle cooperative driving systems, inherent differences in powertrain dynamics, response lags, and external disturbances lead to vehicle-level dynamic heterogeneity, which directly constrains cooperative control performance. To address uncertain vehicle dynamics in heterogeneous platoons, Gao et al. [5] developed a robust control strategy to enhance platoon stability in the presence of dynamic uncertainties and communication delays. Viadero-Monasterio et al. [6] combined Kalman filtering with robust control to achieve online estimation and dynamic compensation of road-gradient effects, aerodynamic drag, and rolling resistance. More recently, Ding et al. [7] developed an adaptive sliding mode control scheme incorporating an improved integral compensation mechanism and a neural-network-based adaptive updating strategy to address actuator input delays and unknown time-varying control coefficients in vehicle platoons. Xiao et al. [8] retained engine inertial lag in a third-order vehicle model and co-designed dynamic event-triggered scheduling and platooning control under random communication topologies and different spacing policies. Their formulation connects powertrain response lags with network-dependent platoon coordination. These studies demonstrate the importance of accounting for vehicle-level dynamic uncertainties in cooperative platoon control. However, platoon performance is also strongly affected by uncertainties in the communication network, where signal blockage, multipath fading, and communication interference may cause random disconnections and reconnections of inter-vehicle communication links.

The inter-vehicle information-exchange relationships determine the propagation paths of state information throughout the platoon and are therefore key factors affecting consensus convergence and platoon stability [4]. Early studies on vehicle platoon control were primarily based on fixed communication topologies, in which predefined information-flow structures were employed to achieve platoon tracking and facilitate stability analysis [9,10]. Subsequently, Li et al. [11] extended these studies to directed topologies and networks with bounded eigenvalues, thereby providing a theoretical foundation for distributed control under more complex information-exchange conditions. However, these methods rely heavily on global graph-spectrum information and have difficulty dynamically characterizing the uncertainty caused by random communication-link disconnections and reconnections in complex physical environments.

To address this limitation, Wen et al. [12] and Ding et al. [13] modeled the topology switching process as a continuous-time Markov chain (CTMC). This approach has gradually become an effective paradigm for handling uncertain communication environments and provides a useful tool for cooperative vehicle platoon control under uncertain communication topologies. Xu et al. [14] combined a Markovian topology model with Lyapunov–Krasovskii analysis and LMI-based $H_{\infty }$ control for heterogeneous platoons with information delays and disturbances, linking stochastic internal stability to an $L_{2}$ string-stability criterion. Building on this framework, He et al. further integrated distributed model predictive control to achieve cooperative control of heterogeneous vehicle platoons under randomly switching topologies [15]. Que et al. [16] subsequently combined a distributed adaptive observer with model predictive control under continuous-time Markovian switching networks, addressing mean-square consensus and explicit predecessor–follower string-stability constraints. Fan et al. [17] proposed a consensus-based control scheme for platoons with time-varying delays and random switching topologies, where consensus and string stability are analyzed using Lyapunov–Krasovskii methods. In addition, Zuo et al. [18] introduced an event-triggered platooning protocol for nonlinear vehicles under Markovian switching communication topologies and external disturbances, thereby reducing unnecessary communication updates while ensuring closed-loop stability. Despite the aforementioned progress, comprehensive research addressing stochastic topology switching, channels with link-dependent heterogeneous time delays, and realistic vehicle-following dynamics requires further exploration. Moreover, in practical communication scenarios, information transmission is affected not only by stochastic variations in spatial connectivity but also by significant time delays that cannot be overlooked.

Information-transmission delays introduce time lags, which can trigger oscillatory responses and compromise the string stability of vehicle platoons. When a follower vehicle receives outdated state information from the lead vehicle, its controller may react based on stale data, thereby amplifying tracking errors or even destabilizing the closed-loop system [19]. Existing research on communication delays in vehicle platoons has addressed scenarios involving time-varying delays, heterogeneous delays, and nonlinear vehicle-following interactions [20,21]. Shen et al. [22] derived mean-square stability conditions and distributed controllers for communication delays combined with identical or non-identical packet-dropout rates, clarifying the joint roles of delay, packet loss, and information-flow topology. Regarding stochastic communication delays, Zhao et al. [23] proposed a cloud-based, delay-dependent predictive control method and validated its effectiveness in terms of asymptotic stability and string stability through experiments with actual vehicle platoons. Meanwhile, to reduce the complexity of closed-loop stability analysis, some researchers have focused on delay disparities among vehicles to better represent real-world communication environments [24,25,26]. However, in scenarios involving stochastic topology switching, the connectivity status of communication links shifts with the topology, and transmission delays vary across links and fluctuate over time. This dual uncertainty makes it difficult for the aforementioned methods to accurately characterize actual conditions. Consequently, approximating multiple communication delays with a single, unified value may obscure the temporal heterogeneity across channels and lead to overly conservative stability analysis.

The stochastic character of communication also affects how platoon stability is assessed. Acciani et al. [27] designed cooperative adaptive cruise control to promote string stability of the average error dynamics while reducing trajectory variance under message losses. Gordon et al. [28] established necessary and sufficient mean-square stability conditions for heterogeneous platoons with Bernoulli data losses and compensation strategies, distinguishing convergence of tracking-error moments from string stability. These results provide relevant context for separating consensus convergence from disturbance propagation in stochastic platoons.

Existing studies have jointly addressed switching topologies and communication delays [14,17,24]. Among these, Yu et al. [24] considered a closely related problem, but their asymptotic stability analysis imposes an average-dwell-time restriction and uses a common delay interval and delay-rate bound across communication links. For heterogeneous third-order vehicle platoons under continuous-time Markovian topology switching, the present study combines delayed neighboring-state information with mode-dependent pinning on leader-relative errors to formulate a closed-loop error model with separate communication-delay channels, while retaining vehicle-following interactions and powertrain inertial lags. Both studies employ Lyapunov–Krasovskii methods and matrix inequality techniques; the present mode-dependent criterion explicitly incorporates the Markov transition rates and link-specific delay and delay-rate bounds to establish nominal mean-square asymptotic stability. This formulation allows heterogeneous delay configurations to be assessed without assigning the larger delay bound of an individual link to all remaining links. Its analytical value is illustrated by the controlled delay-bound comparison in Section 4.

The main contributions are summarized as follows:

- A closed-loop error model is established for heterogeneous third-order vehicle platoons under continuous-time Markovian topology switching, with separate communication-delay channels that retain link-specific delay and delay-rate bounds.
- A distributed cooperative control protocol combines delayed neighboring-state information with mode-dependent leader pinning to regulate leader-relative tracking errors. The protocol incorporates CTH spacing and FVD interactions for third-order vehicles with heterogeneous powertrain inertial lags.
- A sufficient mean-square asymptotic stability criterion is derived for the nominal closed-loop error system using a mode-dependent Lyapunov–Krasovskii functional and the Schur complement. The resulting LMIs provide a computational stability test for prescribed controller parameters and heterogeneous communication-delay bounds. Under the stated assumptions, the same certificate further establishes mean-square $L_{\infty }$ bounds on the propagation of spacing, velocity, and acceleration errors along the platoon.

The remainder of this paper is organized as follows. Section 2 presents the vehicle platoon model, communication topology, and consensus control objectives. Section 3 develops the distributed control strategy and derives the corresponding stability conditions. Section 4 presents the simulation results and discussion on cooperative tracking and car-following safety under the Extra-Urban Driving Cycle (EUDC). Finally, Section 5 concludes the paper and outlines future research directions.

Notation: Throughout this paper, R denotes the field of real numbers, $R^{n}$ denotes the *n*-dimensional real vector space, and $R^{m\times n}$ denotes the set of m×n real matrices. For an arbitrary matrix *X*, $X^{T}$ denotes its transpose. If *X* is symmetric, X>0 and X<0 indicate that *X* is positive definite and negative definite, respectively. $I_{n}$ denotes the *n*-dimensional identity matrix. The operator diag{·} denotes a diagonal or block-diagonal matrix constructed from the specified elements or matrices, whereas col{·} denotes a column vector formed by vertically stacking the specified vectors. Moreover, E{·} denotes mathematical expectation, Pr{·} denotes the probability of an event, and $\mathrm{He}\left\{X\right\}=X+X^{T}$ denotes the symmetric part of *X*. The notation ∥·∥ denotes the Euclidean norm, and ∗ represents the symmetric block of a matrix. Unless otherwise specified, all matrices are assumed to have compatible dimensions.

## 2. Preliminaries and Problem Formulation

### 2.1. Vehicle Platoon Dynamics Model

**Figure 1:**
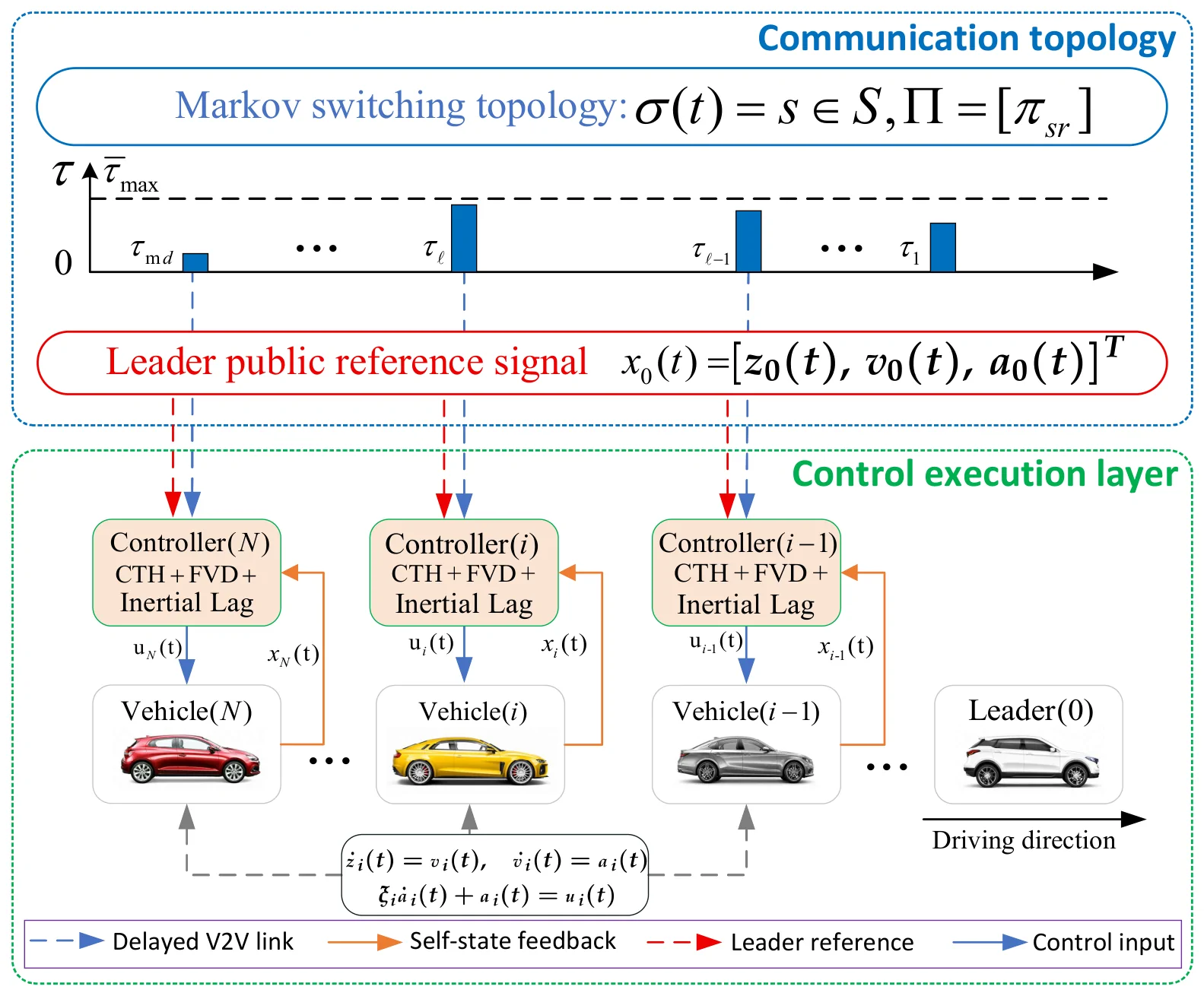
Schematic of the proposed distributed control system.

Consider a connected vehicle platoon consisting of one leader and *N* followers. For the subsequent formulation, define the node set as V={0,1,…,N}, where 0 denotes the leader, and $V_{F}=\left\{1,2,\ldots ,N\right\}$ denotes the set of followers. Let $z_{i}\left(t\right)$, $v_{i}\left(t\right)$, and $a_{i}\left(t\right)$ represent the longitudinal position, velocity, and acceleration of vehicle *i*, respectively. Each follower uses its own state, delayed neighbor information, and the leader reference to track the platoon while maintaining a safe inter-vehicle distance. Figure 1 illustrates the distributed cooperative control architecture adopted in this study.

In practice, vehicle longitudinal dynamics are nonlinear because of aerodynamic drag, rolling resistance, and drivetrain effects. The communication topology is also subject to random variation. Therefore, this study adopts a third-order linear node model obtained through exact feedback linearization to describe the longitudinal motion of each vehicle, while explicitly accounting for heterogeneous transient response lags in vehicle dynamics. The longitudinal dynamics of vehicle *i* are [24,25]

(1) $$\left\{\begin{array}{c} {\dot{z}}_{i}\left(t\right)=v_{i}\left(t\right)\\ {\dot{v}}_{i}\left(t\right)=a_{i}\left(t\right)\\ \xi_{i}{\dot{a}}_{i}\left(t\right)+a_{i}\left(t\right)=u_{i}\left(t\right), \end{array}\right.$$

where $\xi_{i}>0$ denotes the powertrain inertial-lag time constant of vehicle *i*, which characterizes the physical response delay of the vehicle propulsion and braking systems; $u_{i}\left(t\right)$ represents the control input.

### 2.2. Markov-Switching-Based Communication Topology

A directed augmented graph $\bar{G}$ is employed to model the communication topology formed by the leader and the followers [24]. For the subgraph G consisting of *N* following vehicles, its associated adjacency matrix is defined as $A=\left[\alpha_{ij}\right]\in R^{N\times N}$, where $\alpha_{ij}>0$ if follower *i* can receive information from vehicle *j*; otherwise, $\alpha_{ij}=0$. Self-communication is excluded, i.e., $\alpha_{ii}=0$. To characterize the cooperative influence of the leader on the followers, the pinning indicator $\alpha_{i0}$ is introduced. Specifically, $\alpha_{i0}>0$ if the leader state is directly incorporated into the control input of follower *i*; otherwise, $\alpha_{i0}=0$.

In real driving environments, inter-vehicle communication links are subject to random disconnections and reconnections owing to signal interference and physical-distance constraints. To characterize such network uncertainties, the stochastic switching process of the communication topology is modeled as a continuous-time Markov chain (CTMC), denoted by σ(t). Let S={1,2,…,q} represent the topology-mode set. For each communication-topology mode s∈S, the corresponding adjacency matrix is defined as $A_{s}=\left[\alpha_{ij}^{\left(s\right)}\right]$. Since the topology mode activated at time *t* is determined by the Markov chain σ(t), the corresponding active adjacency matrix is denoted by $A_{\sigma (t)}=\left[\alpha_{ij}^{\left(\sigma (t)\right)}\right]$.

**Remark 1.** 
*The symbols $\alpha_{ij}$ and $\alpha_{i0}$ are used solely to indicate the connectivity status at the topological level of the cyber-physical network, and are not equivalent to the actual control gain weights in the subsequent distributed cooperative control law. The dynamic state of the leader vehicle serves only as a global common reference signal in the construction of the local tracking errors.*

The evolution of the stochastic switching process is governed by the transition-rate matrix $\Pi =\left[\pi_{sr}\right]\in R^{q\times q}$ [12,13]. For a sufficiently small time increment Δt>0, the state transition probabilities are defined as

(2) $$\begin{array}{cc} \; & \mathrm{Pr}\;\left\{\sigma (t+\Delta t)=r\;|\;\sigma (t)=s\right\}\\ \; & \;=\left\{\begin{array}{cc} \pi_{sr}\Delta t+o\left(\Delta t\right), & r\neq s\\ 1+\pi_{ss}\Delta t+o\left(\Delta t\right), & r=s \end{array}\right., \end{array}$$

where $\lim_{\Delta t\to 0^{+}}o\left(\Delta t\right)/\Delta t=0$, $\pi_{sr}\geq 0$ (r≠s) denotes the transition rate from mode *s* to mode *r*, and the diagonal entries satisfy $\pi_{ss}=-\sum_{\begin{array}{c} r=1\\ r\neq s \end{array}}^{q}\pi_{sr}$. Under the CTMC, the inter-vehicle communication weights and leader-pinning indicators become mode-dependent.

### 2.3. Consensus Control Objectives

This study aims to drive all follower states to asymptotic synchronization with the leader while maintaining the desired safe inter-vehicle distance under communication delays and external disturbances [29].

Additionally, the constant time headway (CTH) policy is adopted in this study [30]. Let the leader state serve as the global reference. The desired position offset of the *i*th follower vehicle relative to the leader is defined as $d_{i0}\left(t\right)=-h_{i0}v_{0}\left(t\right)$, where $h_{i0}>0$ is the prescribed constant time headway. Since the follower vehicles are behind the leader vehicle, their desired positions are negative offsets along the driving direction relative to the leader. Under this spacing policy, the local tracking-error vector of follower *i* is defined as [24,31]

(3) $$\left\{\begin{array}{c} e_{z,i}\left(t\right)=z_{i}\left(t\right)-z_{0}\left(t\right)-d_{i0}\\ e_{v,i}\left(t\right)=v_{i}\left(t\right)-v_{0}\left(t\right)\\ e_{a,i}\left(t\right)=a_{i}\left(t\right)-a_{0}\left(t\right). \end{array}\right.$$

The consensus-control objective is

(4) $$\left\{\begin{array}{c} \lim_{t\to \infty }∥e_{z,i}\left(t\right)∥=0\\ \lim_{t\to \infty }∥e_{v,i}\left(t\right)∥=0\\ \lim_{t\to \infty }∥e_{a,i}\left(t\right)∥=0. \end{array}\right.$$

## 3. Control Strategy and Stability Analysis

### 3.1. Distributed Cooperative Control Strategy

Based on the objectives of consensus control and the Markov-switching communication topology, the distributed cooperative control protocol is developed by considering time-varying delays and communication uncertainty. Let $k_{ij}>0$ denote the fixed communication edge weight, and $\alpha_{ij}^{\left(s\right)}$ be the adjacency matrix element when the system is in the topology mode σ(t)=s. Thus, the effective communication coupling weight $\kappa_{ij}^{\left(s\right)}$ for the *i*th follower vehicle when receiving information from its neighbor *j* is defined as

(5) $$\kappa_{ij}^{\left(s\right)}=k_{ij}\alpha_{ij}^{\left(s\right)}.$$

Considering the driving interaction behavior, this study also introduces the full velocity difference (FVD) vehicle-following mechanism, combined with the global pinning strategy of the leader vehicle [32]. To couple network connectivity with physical vehicle-following behavior, the equivalent position-coupling coefficient between followers is defined as

(6) $$c_{ij}^{\left(s\right)}=\left(\delta \phi_{ij}+1\right)\kappa_{ij}^{\left(s\right)},$$

where δ represents the FVD interaction adjustment parameter; and $\phi_{ij}$ means the local linearization coefficients for inter-follower interaction. Similarly, the equivalent pinning coupling coefficient from the leader vehicle to follower vehicle *i* is defined as $c_{i0}^{\left(s\right)}=\left(\delta \phi_{i0}+1\right)\kappa_{i0}^{\left(s\right)}$.

In practical vehicle-to-vehicle (V2V) communication environments, information transmission inevitably suffers from time-varying delays. For the state information of neighbor vehicle *j* received by vehicle *i*, both the position error and the velocity error correspond to the delayed instant, and are respectively denoted as $e_{z,j}\left(t-\tau_{ij}\left(t\right)\right)$ and $e_{v,j}\left(t-\tau_{ij}\left(t\right)\right)$, where $\tau_{ij}\left(t\right)$ is the communication delay from vehicle *j* to vehicle *i*. Consequently, based on the aforesaid objectives of consensus, the distributed control law for follower *i* is designed as follows:

(7) $$\begin{array}{cc} u_{i}\left(t\right)=-\{\; & \sum_{j=1}^{N}\kappa_{ij}^{\left(s\right)}[\left(\delta \phi_{ij}+1\right)\left(e_{z,i}\left(t\right)-e_{z,j}\left(t-\tau_{ij}\left(t\right)\right)\right)\\ \; & \;\;+\left(\mu -\delta \right)e_{v,i}\left(t\right)-\mu e_{v,j}\left(t-\tau_{ij}\left(t\right)\right)]\\ \; & +\kappa_{i0}^{\left(s\right)}\left[\left(\delta \phi_{i0}+1\right)e_{z,i}\left(t\right)+\left(\mu -\delta \right)e_{v,i}\left(t\right)\right]\\ \; & +\gamma_{i}e_{z,i}\left(t\right)+\rho_{i}\gamma_{i}\left(e_{v,i}\left(t\right)+e_{a,i}\left(t\right)\right)\}, \end{array}$$

where $\gamma_{i}>0$ denotes the baseline feedback gain; $\rho_{i}>0$ represents the dynamic weighting coefficient assigned to the velocity and acceleration channels relative to the position channel; and μ is the velocity difference adjustment parameter. $\sum_{j=1}^{N}\kappa_{ij}^{\left(s\right)}\left[\cdot \right]$ is the delayed neighboring-vehicle coupling term, which characterizes the cooperative adjustment of follower *i* based on the delayed state information received from its neighboring vehicle *j*; $\kappa_{i0}^{\left(s\right)}\left[\cdot \right]$ represents the leader-pinning term, describing the direct influence of the leader’s reference state on follower *i*; and $\gamma_{i}e_{z,i}\left(t\right)+\rho_{i}\gamma_{i}\left(e_{v,i}\left(t\right)+e_{a,i}\left(t\right)\right)$ denotes the local error-feedback term, which strengthens the corrective action on the position, velocity, and acceleration errors of the vehicle *i*.

By separately expanding the current-state terms and the delayed neighboring-vehicle state terms in (7), the proposed distributed cooperative control law is therefore expressed as

(8) $$\begin{array}{cc} u_{i}\left(t\right)= & -\left[\gamma_{i}+\sum_{j=1}^{N}c_{ij}^{\left(s\right)}+c_{i0}^{\left(s\right)}\right]e_{z,i}\left(t\right)\\ \; & -\left[\gamma_{i}\rho_{i}+\left(\mu -\delta \right)\left(\sum_{j=1}^{N}\kappa_{ij}^{\left(s\right)}+\kappa_{i0}^{\left(s\right)}\right)\right]e_{v,i}\left(t\right)\\ \; & -\gamma_{i}\rho_{i}e_{a,i}\left(t\right)+\sum_{j=1}^{N}c_{ij}^{\left(s\right)}e_{z,j}\left(t-\tau_{ij}\left(t\right)\right)\\ \; & +\sum_{j=1}^{N}\mu \kappa_{ij}^{\left(s\right)}e_{v,j}\left(t-\tau_{ij}\left(t\right)\right). \end{array}$$

### 3.2. Closed-Loop System Dynamics

To isolate the nominal delay-and-switching dynamics used by the LMI synthesis, we first consider the constant-leader case [24,33]. In that case, the desired position offset under the CTH policy is $d_{i0}=-h_{i0}v_{0}$ and ${\dot{d}}_{i0}=0$. When $a_{0}\left(t\right)=0$, it follows that $e_{a,i}\left(t\right)=a_{i}\left(t\right)$ and ${\dot{e}}_{a,i}\left(t\right)={\dot{a}}_{i}\left(t\right)$, which, combined with (1), yields the acceleration error dynamics of follower vehicle *i* under the constant-leader assumption as $\xi_{i}{\dot{e}}_{a,i}\left(t\right)=u_{i}\left(t\right)-e_{a,i}\left(t\right)$. Combining this result with (8) further yields

(9) $$\;\begin{array}{cc} \xi_{i}{\dot{e}}_{a,i}\left(t\right)= & -\left[\gamma_{i}+\sum_{j=1}^{N}c_{ij}^{\left(s\right)}+c_{i0}^{\left(s\right)}\right]e_{z,i}\left(t\right)\\ \; & -\left[\gamma_{i}\rho_{i}+\left(\mu -\delta \right)\left(\sum_{j=1}^{N}\kappa_{ij}^{\left(s\right)}+\kappa_{i0}^{\left(s\right)}\right)\right]e_{v,i}\left(t\right)\\ \; & -\left(1+\gamma_{i}\rho_{i}\right)e_{a,i}\left(t\right)+\sum_{j=1}^{N}c_{ij}^{\left(s\right)}e_{z,j}\left(t-\tau_{ij}\left(t\right)\right)\\ \; & +\sum_{j=1}^{N}\mu \kappa_{ij}^{\left(s\right)}e_{v,j}\left(t-\tau_{ij}\left(t\right)\right). \end{array}$$

Define ${\left[M_{1,s}\right]}_{ii}=\gamma_{i}+\sum_{j=1}^{N}c_{ij}^{\left(s\right)}+c_{i0}^{\left(s\right)}$, ${\left[M_{2,s}\right]}_{ii}=\gamma_{i}\rho_{i}+\left(\mu -\delta \right)\left(\sum_{j=1}^{N}\kappa_{ij}^{\left(s\right)}+\kappa_{i0}^{\left(s\right)}\right)$, and ${\left[M_{3}\right]}_{ii}=1+\gamma_{i}\rho_{i}$. Then, (9) can be rewritten as

(10) $$\begin{array}{cc} {\dot{e}}_{a,i}\left(t\right)= & -\frac{1}{\xi_{i}}{\left[M_{1,s}\right]}_{ii}e_{z,i}\left(t\right)-\frac{1}{\xi_{i}}{\left[M_{2,s}\right]}_{ii}e_{v,i}\left(t\right)\\ \; & -\frac{1}{\xi_{i}}{\left[M_{3}\right]}_{ii}e_{a,i}\left(t\right)+\frac{1}{\xi_{i}}\sum_{j=1}^{N}c_{ij}^{\left(s\right)}e_{z,j}\left(t-\tau_{ij}\left(t\right)\right)\\ \; & +\frac{1}{\xi_{i}}\sum_{j=1}^{N}\mu \kappa_{ij}^{\left(s\right)}e_{v,j}\left(t-\tau_{ij}\left(t\right)\right). \end{array}$$

Define global error vectors as $e_{z}\left(t\right)={\left[e_{z,1}\left(t\right),e_{z,2}\left(t\right),\ldots ,e_{z,N}\left(t\right)\right]}^{T}$, $e_{v}\left(t\right)={\left[e_{v,1}\left(t\right),e_{v,2}\left(t\right),\ldots ,e_{v,N}\left(t\right)\right]}^{T}$, and $e_{a}\left(t\right)={\left[e_{a,1}\left(t\right),e_{a,2}\left(t\right),\ldots ,e_{a,N}\left(t\right)\right]}^{T}$. For the nominal case considered here, it follows from (3) that the global error vector satisfies ${\dot{e}}_{z}\left(t\right)=e_{v}\left(t\right)$ and ${\dot{e}}_{v}\left(t\right)=e_{a}\left(t\right)$. The forced case with $a_{0}\left(t\right)\neq 0$ is treated in Section 3.4. Furthermore, let $1_{N}={\left(1\;1\;\ldots \;1\right)}^{T}\in R^{N}$. The row-sum matrices for the velocity and position channels are defined, respectively, as $D_{K,s}=\mathrm{diag}\left(K_{s}1_{N}+\kappa_{0,s}\right)$ and $D_{c,s}=\mathrm{diag}\left(C_{s}1_{N}+c_{0,s}\right)$. Hence, we have

(11) $$\;\begin{array}{cc} {\dot{e}}_{a}\left(t\right)= & -TM_{1,s}e_{z}\left(t\right)-TM_{2,s}e_{v}\left(t\right)-TM_{3}e_{a}\left(t\right)\\ \; & +\sum_{i=1}^{N}\sum_{j=1}^{N}Tc_{ij}^{\left(s\right)}E_{ij}e_{z}\left(t-\tau_{ij}\left(t\right)\right)\\ \; & +\sum_{i=1}^{N}\sum_{j=1}^{N}T\mu \kappa_{ij}^{\left(s\right)}E_{ij}e_{v}\left(t-\tau_{ij}\left(t\right)\right), \end{array}$$

where

$$\left\{\begin{array}{cc} M_{1,s} & =\Gamma +D_{c,s}=\mathrm{diag}{\left\{\gamma_{i}+\sum_{j=1}^{N}c_{ij}^{\left(s\right)}+c_{i0}^{\left(s\right)}\right\}}_{i=1}^{N}\\ M_{2,s} & =\Gamma R_{\rho }+\left(\mu -\delta \right)D_{K,s}\\ & =\mathrm{diag}{\left\{\gamma_{i}\rho_{i}+\left(\mu -\delta \right)\left(\sum_{j=1}^{N}\kappa_{ij}^{\left(s\right)}+\kappa_{i0}^{\left(s\right)}\right)\right\}}_{i=1}^{N}\\ M_{3} & =I_{N}+\Gamma R_{\rho }=\mathrm{diag}{\left\{1+\gamma_{i}\rho_{i}\right\}}_{i=1}^{N}, \end{array}\right.$$

$T=\mathrm{diag}\{1/\xi_{1},\ldots ,1/\xi_{N}\}$ is the diagonal matrix of inverse powertrain time constants; $\Gamma =\mathrm{diag}\{\gamma_{1},\ldots ,\gamma_{N}\}$ is the baseline feedback matrix; $R_{\rho }=\mathrm{diag}\left\{\rho_{1},\ldots ,\rho_{N}\right\}$ is the dynamic adjustment matrix; $K_{s}={\left[\kappa_{ij}^{\left(s\right)}\right]}_{N\times N}$ is the topological coupling matrix; and $C_{s}={\left[c_{ij}^{\left(s\right)}\right]}_{N\times N}$, $\kappa_{0,s}={\left[\kappa_{10}^{\left(s\right)},\ldots ,\kappa_{N0}^{\left(s\right)}\right]}^{T}$, and $c_{0,s}={\left[c_{10}^{\left(s\right)},\ldots ,c_{N0}^{\left(s\right)}\right]}^{T}$.

Let $e_{i}\in R^{N}$ denote the *i*th standard basis vector, and define the link selection matrix $E_{ij}=e_{i}e_{j}^{T}\in R^{N\times N}$. For any global error vector, we have $E_{ij}e_{z}\left(t\right)=e_{i}e_{z,j}\left(t\right)$ and $E_{ij}e_{v}\left(t\right)=e_{i}e_{v,j}\left(t\right)$. To uniformly describe the heterogeneous time-varying delays of different communication links, it is assumed that there are $m_{d}$ possible communication links in the actual communication network, which are sequentially indexed by $\ell \in \{1,2,\ldots ,m_{d}\}$. If the ℓth communication link corresponds to the information transmission from the node *j* to the node *i*, then the time-varying delay of this link is denoted as $\tau_{\ell }\left(t\right)=\tau_{ij}\left(t\right)$. Consequently, the delayed coupling term corresponding to the ℓth communication link can be expressed as $Tc_{ij}^{\left(s\right)}E_{ij}e_{z}\left(t-\tau_{\ell }\left(t\right)\right)+T\mu \kappa_{ij}^{\left(s\right)}E_{ij}e_{v}\left(t-\tau_{\ell }\left(t\right)\right)$. The delayed position and velocity errors of neighboring vehicles enter ${\dot{e}}_{a}\left(t\right)$ through the control input, rather than directly entering ${\dot{e}}_{z}\left(t\right)$ or ${\dot{e}}_{v}\left(t\right)$.

Consequently, by defining the global augmented-state vector as $\zeta \left(t\right)={\left[e_{z}^{T}\left(t\right),e_{v}^{T}\left(t\right),e_{a}^{T}\left(t\right)\right]}^{T}$ and defining the delayed augmented state vector for the ℓth communication link as $\zeta \left(t-\tau_{\ell }\left(t\right)\right)={\left[e_{z}^{T}\left(t-\tau_{\ell }\left(t\right)\right),e_{v}^{T}\left(t-\tau_{\ell }\left(t\right)\right),e_{a}^{T}\left(t-\tau_{\ell }\left(t\right)\right)\right]}^{T}$, we obtain the closed-loop system dynamics of the vehicle platoon under topology mode σ(t)=s as follows:

(12) $$\dot{\zeta }\left(t\right)=A_{0,\sigma (t)}\zeta \left(t\right)+\sum_{\ell =1}^{m_{d}}A_{\ell ,\sigma (t)}\zeta \left(t-\tau_{\ell }\left(t\right)\right),$$

where

$$A_{0,\sigma (t)=s}=\left[\begin{array}{ccc} 0_{N\times N} & I_{N} & 0_{N\times N}\\ 0_{N\times N} & 0_{N\times N} & I_{N}\\ -TM_{1,s} & -TM_{2,s} & -TM_{3} \end{array}\right],$$

$$A_{\ell ,\sigma (t)=s}=\left[\begin{array}{ccc} 0_{N\times N} & 0_{N\times N} & 0_{N\times N}\\ 0_{N\times N} & 0_{N\times N} & 0_{N\times N}\\ Tc_{ij}^{\left(s\right)}E_{ij} & T\mu \kappa_{ij}^{\left(s\right)}E_{ij} & 0_{N\times N} \end{array}\right].$$

The matrix $A_{0,s}$ represents the delay-free dynamics formed by each vehicle’s own error feedback, leader pinning, and the current-state terms in the control law, whereas $A_{\ell ,s}$ represents the delayed coupling effect from neighboring vehicles via the ℓth communication link. If the ℓth communication link is disconnected under topology mode *s*, then $\kappa_{ij}^{\left(s\right)}=0$ and $c_{ij}^{\left(s\right)}=0$, and hence $A_{\ell ,s}=0$.

### 3.3. Stability Analysis

Let ${\bar{\tau }}_{\ell }>0$ denote the upper bound of the ℓth heterogeneous delay channel, and define the global maximum delay bound as ${\bar{\tau }}_{\max}=\max_{\ell \in \{1,2,\ldots ,m_{d}\}}{\bar{\tau }}_{\ell }$. Let $C=C(\left[-{\bar{\tau }}_{\max},0\right],R^{3N})$ denote the space of continuous functions containing the initial history functions of the closed-loop system [34]. For the proposed system (12), the state-history segment at time *t* is defined as

(13) $$\zeta_{t}\left(\theta \right)=\zeta \left(t+\theta \right),\;\theta \in \left[-{\bar{\tau }}_{\max},0\right].$$

The initial history function of the proposed system is specified as

(14) $$\zeta_{0}\left(\theta \right)=\phi \left(\theta \right),\;\phi \in C.$$

Using this initial history function, the stability of the Markovian switching time-delay closed-loop system is defined below.

**Definition** **1** ([35])**.*** Consider the closed-loop system* (12) *of the vehicle platoon. For any initial topology mode σ(0)∈S and any initial history function ϕ∈C, if the corresponding state trajectory satisfies*

(15) $$\lim_{t\to \infty }E\left\{{∥\zeta \left(t\right)∥}^{2}\right\}=0,$$

*then the proposed closed-loop system is said to be mean-square asymptotically stable.*

Since the state evolution of system (12) is jointly governed by the continuous-time dynamics and the Markovian switching process, the following mathematical lemmas are introduced to derive sufficient conditions for its mean-square asymptotic stability.

**Lemma** **1** (Jensen’s Integral Inequality [36,37])**.** *For any given symmetric positive-definite matrix R, scalar h>0, and vector-valued function ω: $\left[-h,0\right]\to R^{n}$ such that the following integrals are well defined, it holds that:*

(16) $$-h\int_{t-h}^{t}\omega^{T}\left(s\right)R\omega \left(s\right)\;ds\leq -{\left[\int_{t-h}^{t}\omega \left(s\right)\;ds\right]}^{T}R\left[\int_{t-h}^{t}\omega \left(s\right)\;ds\right].$$

**Lemma** **2** (Schur Complement Lemma [38])**.** *Consider a given symmetric matrix $S=\left[\begin{array}{cc} S_{11} & S_{12}\\ {}* & S_{22} \end{array}\right]$, where $S_{11}$ and $S_{22}$ are square matrices. Then, the following three conditions are equivalent:*

$$\begin{array}{cc} \left(1\right) & S<0,\\ \left(2\right) & S_{11}<0\;\mathrm{and}\;S_{22}-S_{12}^{T}S_{11}^{-1}S_{12}<0,\\ \left(3\right) & S_{22}<0\;\mathrm{and}\;S_{11}-S_{12}S_{22}^{-1}S_{12}^{T}<0. \end{array}$$

With the aim of converting the closed-loop system (12) into a compact LMI-compatible form, the actual time-varying delayed state associated with the ℓth heterogeneous delay channel and the state at the delay upper bound are respectively defined as $\zeta_{\ell }\left(t\right)=\zeta \left(t-\tau_{\ell }\left(t\right)\right)$ and ${\bar{\zeta }}_{\ell }\left(t\right)=\zeta \left(t-{\bar{\tau }}_{\ell }\right)$. Accordingly, the global augmented state vector is constructed as

(17) $$\chi \left(t\right)=\mathrm{col}\left\{\zeta \left(t\right),\zeta_{1}\left(t\right),\ldots ,\zeta_{m_{d}}\left(t\right),{\bar{\zeta }}_{1}\left(t\right),\ldots ,{\bar{\zeta }}_{m_{d}}\left(t\right)\right\}.$$

Based on the augmented state vector defined in (17), we have $\zeta \left(t\right)=E_{0}\chi \left(t\right)$, $\zeta_{\ell }\left(t\right)=E_{\ell }\chi \left(t\right)$, and ${\bar{\zeta }}_{\ell }\left(t\right)={\bar{E}}_{\ell }\chi \left(t\right)$, where $E_{0}$, $E_{\ell }$, and ${\bar{E}}_{\ell }$ are respectively expressed as

(18) $$\left\{\begin{array}{cc} E_{0} & =\left[\begin{array}{cccccc} I_{3N}\; & 0_{3N\times 3N}\; & \ldots \; & 0_{3N\times 3N}\; & \ldots \; & 0_{3N\times 3N} \end{array}\right]\\ E_{\ell } & =\left[\begin{array}{ccccccc} 0_{3N\times 3N}\; & \ldots \; & \underset{(\ell +1)\text{-}\mathrm{th}\;\mathrm{block}}{\underset{︸}{I_{3N}}}\; & \ldots \; & 0_{3N\times 3N}\; & \ldots \; & 0_{3N\times 3N} \end{array}\right]\\ {\bar{E}}_{\ell } & =\left[\begin{array}{ccccccc} 0_{3N\times 3N}\; & \ldots \; & 0_{3N\times 3N}\; & \ldots \; & \underset{(m_{d}+\ell +1)\text{-}\mathrm{th}\;\mathrm{block}}{\underset{︸}{I_{3N}}}\; & \ldots \; & 0_{3N\times 3N} \end{array}\right]. \end{array}\right.$$

Based on the components of the augmented state vector χ(t), and since the upper-bound delayed states ${\bar{\zeta }}_{\ell }\left(t\right)$ are absent from (12), their associated coefficient blocks vanish. With $F_{s}$ defined as $F_{s}=\left[A_{0,s}\;A_{1,s}\;\cdots \;A_{m_{d},s}\;0\;\ldots \;0\right]$, it follows that

(19) $$\begin{array}{cc} F_{s}\chi \left(t\right)\; & =A_{0,s}\zeta \left(t\right)+\sum_{\ell =1}^{m_{d}}A_{\ell ,s}\zeta_{\ell }\left(t\right)\\ \; & =A_{0,s}\zeta \left(t\right)+\sum_{\ell =1}^{m_{d}}A_{\ell ,s}\zeta \left(t-\tau_{\ell }\left(t\right)\right). \end{array}$$

It follows from (12) and (19) that

(20) $$\dot{\zeta }\left(t\right)=F_{s}\chi \left(t\right).$$

The stability conditions are then given in Theorem 1.

**Theorem 1.** 
*For each communication-delay channel $\ell =1,2,\ldots ,m_{d}$, suppose that the time-varying communication delay $\tau_{\ell }\left(t\right)$ is continuously differentiable and satisfies $0\leq \tau_{\ell }\left(t\right)\leq {\bar{\tau }}_{\ell }$ and ${\dot{\tau }}_{\ell }\left(t\right)\leq \mu_{\ell }<1$, where ${\bar{\tau }}_{\ell }>0$ and $0\leq \mu_{\ell }<1$ denote the upper bound of time-varying delay and the time delay-rate upper bound of the ℓth communication-delay channel, respectively. If there exist symmetric positive-definite matrices $P_{s},Q_{\ell },R_{\ell }\in R^{3N\times 3N}$ such that, for every topology mode s∈S, the following linear matrix inequality (LMI) holds:*

(21) $$\Omega_{s}=\left[\begin{array}{ccccc} \Omega_{0,s}\; & {\bar{\tau }}_{1}F_{s}^{T}R_{1}\; & {\bar{\tau }}_{2}F_{s}^{T}R_{2}\; & \ldots \; & {\bar{\tau }}_{m_{d}}F_{s}^{T}R_{m_{d}}\\ {}*\; & -{\bar{\tau }}_{1}R_{1}\; & 0\; & \ldots \; & 0\\ {}*\; & *\; & -{\bar{\tau }}_{2}R_{2}\; & \ldots \; & 0\\ \vdots \; & \vdots \; & \vdots \; & \ddots \; & \vdots \\ {}*\; & *\; & *\; & \ldots \; & -{\bar{\tau }}_{m_{d}}R_{m_{d}} \end{array}\right]<0,$$

*where*

$$\begin{array}{cc} \Omega_{0,s}= & \mathrm{He}\left\{E_{0}^{T}P_{s}F_{s}\right\}+E_{0}^{T}\left(\sum_{r=1}^{q}\pi_{sr}P_{r}+\sum_{\ell =1}^{m_{d}}Q_{\ell }\right)E_{0}\\ \; & -\sum_{\ell =1}^{m_{d}}\left(1-\mu_{\ell }\right)E_{\ell }^{T}Q_{\ell }E_{\ell }\\ \; & -\sum_{\ell =1}^{m_{d}}\frac{1}{{\bar{\tau }}_{\ell }}{\left(E_{0}-{\bar{E}}_{\ell }\right)}^{T}R_{\ell }\left(E_{0}-{\bar{E}}_{\ell }\right). \end{array}$$

*Then, the nominal closed-loop system* (12) *is mean-square asymptotically stable.*

**Proof.**  Let $P_{s},Q_{\ell },R_{\ell }\in R^{3N\times 3N}$ be the symmetric positive-definite matrices. When the communication topology operates in mode σ(t)=s, then construct the following multiple-integral Lyapunov–Krasovskii (L-K) functional as

(22) $$V\left(t,\zeta_{t},s\right)=V_{1}\left(t,\zeta \left(t\right),s\right)+V_{2}\left(t,\zeta_{t}\right)+V_{3}\left(t,\zeta_{t}\right),$$

where

(23a) $$V_{1}\left(t,\zeta (t),s\right)=\zeta^{T}\left(t\right)P_{s}\zeta \left(t\right),$$

(23b) $$V_{2}\left(t,\zeta_{t}\right)=\sum_{\ell =1}^{m_{d}}\int_{t-\tau_{\ell }\left(t\right)}^{t}\zeta^{T}\left(\theta \right)Q_{\ell }\zeta \left(\theta \right)\;d\theta ,$$

(23c) $$V_{3}\left(t,\zeta_{t}\right)=\sum_{\ell =1}^{m_{d}}\int_{-{\bar{\tau }}_{\ell }}^{0}\int_{t+\theta }^{t}{\dot{\zeta }}^{T}\left(\eta \right)R_{\ell }\dot{\zeta }\left(\eta \right)\;d\eta \;d\theta .$$

For the joint Markov process $\left(\zeta_{t},\sigma \left(t\right)\right)$, the weak infinitesimal generator L associated with the L-K functional is defined as follows:

(24) $$\begin{array}{cc} LV(t,\zeta_{t},s)= & \lim_{\Delta t\to 0^{+}}\frac{1}{\Delta t}[E\{V(t+\Delta t,\zeta_{t+\Delta t},\\ \; & \;\sigma \left(t+\Delta t\right))|\zeta_{t},\sigma \left(t\right)=s\}-V\left(t,\zeta_{t},s\right)]. \end{array}$$

Thus, calculating the weak infinitesimal generator along the trajectories of the closed-loop system yields the following expressions for $LV_{1}$ and $LV_{2}$:

(25) $$LV_{1}=2\zeta^{T}\left(t\right)P_{s}\dot{\zeta }\left(t\right)+\zeta^{T}\left(t\right)\left(\sum_{r=1}^{q}\pi_{sr}P_{r}\right)\zeta \left(t\right),$$

(26) $$LV_{2}\leq \sum_{\ell =1}^{m_{d}}\zeta^{T}\left(t\right)Q_{\ell }\zeta \left(t\right)-\sum_{\ell =1}^{m_{d}}\left(1-\mu_{\ell }\right)\zeta_{\ell }^{T}\left(t\right)Q_{\ell }\zeta_{\ell }\left(t\right).$$

Based on Theorem 1, if the delay-variation condition $1-{\dot{\tau }}_{\ell }\left(t\right)\geq 1-\mu_{\ell }>0$ is satisfied, then the quadratic term corresponding to the delayed state remains non-positive. Therefore, by applying Lemma 1 to perform a bounding estimation on the negative definite integral term in $LV_{3}$, we have

(27) $$\begin{array}{cc} LV_{3}= & \sum_{\ell =1}^{m_{d}}{\bar{\tau }}_{\ell }{\dot{\zeta }}^{T}\left(t\right)R_{\ell }\dot{\zeta }\left(t\right)\\ \; & -\sum_{\ell =1}^{m_{d}}\int_{t-{\bar{\tau }}_{\ell }}^{t}{\dot{\zeta }}^{T}\left(\eta \right)R_{\ell }\dot{\zeta }\left(\eta \right)\;d\eta \\ \leq & \sum_{\ell =1}^{m_{d}}{\bar{\tau }}_{\ell }{\dot{\zeta }}^{T}\left(t\right)R_{\ell }\dot{\zeta }\left(t\right)\\ \; & -\sum_{\ell =1}^{m_{d}}\frac{1}{{\bar{\tau }}_{\ell }}{\left[\zeta \left(t\right)-{\bar{\zeta }}_{\ell }\left(t\right)\right]}^{T}R_{\ell }\\ \; & \;\;\times \left[\zeta \left(t\right)-{\bar{\zeta }}_{\ell }\left(t\right)\right]. \end{array}$$

Combining (25) to (27) yields

(28) $$LV\left(t,\zeta_{t},s\right)\leq \chi^{T}\left(t\right)\left[\Omega_{0,s}+F_{s}^{T}\left(\sum_{\ell =1}^{m_{d}}{\bar{\tau }}_{\ell }R_{\ell }\right)F_{s}\right]\chi \left(t\right).$$

According to Lemma 2, the condition $LV(t,\zeta_{t},s)<0$ in (28) is equivalent to the following matrix inequality:

(29) $$\left[\begin{array}{cc} \Omega_{0,s}\; & F_{s}^{T}\left(\sum_{\ell =1}^{m_{d}}{\bar{\tau }}_{\ell }R_{\ell }\right)\\ {}*\; & -\sum_{\ell =1}^{m_{d}}{\bar{\tau }}_{\ell }R_{\ell } \end{array}\right]<0.$$

Based on (29), there exists a scalar ϵ>0 such that $LV\left(t,\zeta_{t},s\right)\leq -\epsilon {∥\zeta \left(t\right)∥}^{2}$. The generator is therefore negative for every nonzero error state, and the invariant set of the closed-loop system contains only the origin. According to the Lyapunov stability theory for stochastic time-delay systems and the LaSalle-type invariance principle for systems with Markovian switching [39,40], the closed-loop system asymptotically converges to zero. If the second moment of the solution is uniformly bounded, then $\lim_{t\to \infty }{E\{∥\zeta \left(t\right)∥}^{2}\}=0$. Consequently, the nominal closed-loop system (12) is mean-square asymptotically stable. This completes the proof. □

### 3.4. ISS Extension for Time-Varying Leader Trajectories

The nominal analysis above is not used as a global assumption for the EUDC simulation. For a time-varying leader, the desired CTH offset remains $d_{i0}\left(t\right)=-h_{i0}v_{0}\left(t\right)$, but its derivative becomes

(30) $${\dot{d}}_{i0}\left(t\right)=-h_{i0}a_{0}\left(t\right).$$

Consequently, the local error dynamics are

(31a) $$\begin{array}{cc} {\dot{e}}_{z,i}\left(t\right) & =e_{v,i}\left(t\right)+h_{i0}a_{0}\left(t\right), \end{array}$$

(31b) $$\begin{array}{cc} {\dot{e}}_{v,i}\left(t\right) & =e_{a,i}\left(t\right), \end{array}$$

(31c) $$\begin{array}{cc} \xi_{i}{\dot{e}}_{a,i}\left(t\right) & =-e_{a,i}\left(t\right)+u_{i}\left(t\right)-a_{0}\left(t\right)-\xi_{i}{\dot{a}}_{0}\left(t\right). \end{array}$$

Equations (31a)–(31c) retain the original feedback law and do not introduce an additional leader feedforward term. The leader motion enters only as an exogenous input, defined as $w\left(t\right)=\mathrm{col}\;\left(a_{0}\left(t\right),{\dot{a}}_{0}\left(t\right)\right)$. Using the global error vector ζ(t) defined in the nominal analysis, the full error system can be written as

(32) $$\dot{\zeta }\left(t\right)=A_{0,\sigma (t)}\zeta \left(t\right)+\sum_{\ell =1}^{m_{d}}A_{\ell ,\sigma (t)}\zeta \left(t-\tau_{\ell }\left(t\right)\right)+B_{w}w\left(t\right),$$

where

$$B_{w}=\left[\begin{array}{cc} h_{10} & 0\\ \vdots & \vdots \\ h_{N0} & 0\\ 0_{N\times 1} & 0_{N\times 1}\\ -T1_{N} & -1_{N} \end{array}\right]\in R^{3N\times 2},$$

and

$$T=\mathrm{diag}\left(\frac{1}{\xi_{1}},\ldots ,\frac{1}{\xi_{N}}\right).$$

The block $-T1_{N}$ corresponds to $a_{0}\left(t\right)$ in the acceleration-error equation, whereas $-1_{N}$ corresponds to ${\dot{a}}_{0}\left(t\right)$. For notational compactness in the proof below, define $R_{\Sigma }=\sum_{\ell =1}^{m_{d}}{\bar{\tau }}_{\ell }R_{\ell }$.

**Corollary 1** (ISS for bounded leader motion)**.** *Suppose that the conditions of Theorem 1 hold, that $a_{0}$ is locally absolutely continuous, and that ${\dot{a}}_{0}\in L_{\infty }\left(\left[0,\infty \right)\right)$. Then the time-varying error system* (32) *is input-to-state stable with respect to w(t). More precisely, there exist constants α>0, β≥1, and γ≥0 such that, for all $0\leq t_{0}\leq t$,*

(33) $$\begin{array}{cc} E\{∥\zeta \left(t\right){∥}^{2}\}\leq & \beta e^{-\alpha (t-t_{0})}\underset{t_{0}-{\bar{\tau }}_{\max}\leq s\leq t_{0}}{\sup}{E\{∥\zeta \left(s\right)∥}^{2}\}\\ \; & +\gamma \underset{t_{0}\leq s\leq t}{\sup}{∥w\left(s\right)∥}^{2}, \end{array}$$

*If, in addition, $a_{0}\left(t\right)\to 0$ and ${\dot{a}}_{0}\left(t\right)\to 0$ as t→∞, then*

(34) $$\lim_{t\to \infty }{E\{∥\zeta \left(t\right)∥}^{2}\}=0.$$

**Proof.**  Since the LMI in Theorem 1 is strict, the Schur complement condition guarantees a scalar ε>0 such that the nominal Lyapunov–Krasovskii certificate satisfies

(35) $$LV_{\mathrm{nom}}\left(t,\zeta_{t},\sigma \left(t\right)\right)\leq -\varepsilon {∥\chi \left(t\right)∥}^{2},$$

along the nominal system (12). In (35), χ(t) is the augmented state defined in (17); hence, the bound is stated with respect to the full delayed-state vector rather than only its current component ζ(t).

Along the forced system (32), write $\dot{\zeta }\left(t\right)=F_{\sigma (t)}\chi \left(t\right)+B_{w}w\left(t\right)$. The weak infinitesimal generator of $V_{1}$ then contains the additional term $2\zeta^{T}\left(t\right)P_{\sigma (t)}B_{w}w\left(t\right)$. Since $V_{2}$ does not depend explicitly on $\dot{\zeta }\left(t\right)$, its generator is unchanged. Substituting the forced dynamics into the leading term of $LV_{3}$ gives

(36) $$\begin{array}{cc} LV\leq & LV_{\mathrm{nom}}+2\zeta^{T}\left(t\right)P_{\sigma (t)}B_{w}w\left(t\right)\\ \; & +2\chi^{T}\left(t\right)F_{\sigma (t)}^{T}R_{\Sigma }B_{w}w\left(t\right)+w^{T}\left(t\right)B_{w}^{T}R_{\Sigma }B_{w}w\left(t\right)\\ \leq & -\frac{\varepsilon }{2}{∥\chi \left(t\right)∥}^{2}+c{∥w\left(t\right)∥}^{2}\\ \leq & -\frac{\varepsilon }{2}{∥\zeta \left(t\right)∥}^{2}+c{∥w\left(t\right)∥}^{2}. \end{array}$$

The additional cross and input terms in the first inequality arise from the complete square ${\left(F_{\sigma (t)}\chi +B_{w}w\right)}^{T}R_{\Sigma }\left(F_{\sigma (t)}\chi +B_{w}w\right)$ in the positive leading term of $LV_{3}$. The additional terms produced inside the negative integral of $LV_{3}$ are non-positive and are therefore discarded when the upper bound is taken. The second inequality follows by applying Young’s inequality $2x^{T}y\leq {\eta ∥x∥}^{2}+\eta^{-1}{∥y∥}^{2}$ separately to the two cross terms with η=ε/4, using ∥ζ(t)∥≤∥χ(t)∥, and choosing c>0 sufficiently large.

The functional *V* is coercive with respect to ${∥\zeta \left(t\right)∥}^{2}$ and has the uniform growth bound required by the Lyapunov–Krasovskii ISS comparison argument for stochastic time-delay systems [41]. The coefficient matrices are mode-dependent but bounded, and $P_{s},Q_{\ell },R_{\ell }$ are uniformly positive definite because the mode set is finite. Consequently, applying the Lyapunov–Krasovskii ISS comparison argument on the interval $\left[t_{0},t\right]$ to (36) yields the exponential ISS estimate (33). At $t_{0}=0$, this estimate recovers the usual bound in terms of the initial history and the input on [0,t].

To establish the vanishing-input limit, let η>0 be arbitrary. Since w(t)→0, there exists $t_{0}\geq 0$ such that ${∥w\left(s\right)∥}^{2}\leq \eta$ for all $s\geq t_{0}$. Applying (33) with this $t_{0}$ and then letting t→∞ gives

$$\underset{t\to \infty }{\lim\sup}{E\{∥\zeta \left(t\right)∥}^{2}\}\leq \gamma \eta .$$

Because η>0 was arbitrary and γ is fixed, the right-hand side can be made arbitrarily small. Therefore,

$$\lim_{t\to \infty }{E\{∥\zeta \left(t\right)∥}^{2}\}=0,$$

which proves (34). This completes the proof. □

### 3.5. Mean-Square $L_{\infty }$ Bounded Propagation

The preceding ISS result establishes bounded tracking with respect to the leader input. We next prove a mean-square $L_{\infty }$ bounded-propagation property along the platoon [13]. Let

(37) $$\delta_{i}\left(t\right)=\mathrm{col}\left(e_{z,i}\left(t\right),e_{v,i}\left(t\right),e_{a,i}\left(t\right)\right)$$

denote the local state-error vector of follower *i*.

**Definition 3.** 
*For a fixed finite platoon size N, the closed-loop platoon has mean-square $L_{\infty }$ bounded propagation if, for every ϵ>0, there exists ϖ>0 such that*

(38) $$\underset{i}{\sup}\underset{-{\bar{\tau }}_{\max}\leq s\leq 0}{\sup}E\{∥\delta_{i}\left(s\right){∥}^{2}\}<\varpi^{2}$$

*implies*

(39) $$\underset{t\geq 0}{\sup}\underset{i}{\sup}E\{∥\delta_{i}\left(t\right){∥}^{2}\}<\epsilon^{2},$$

*where ${\bar{\tau }}_{\max}=\max_{\ell }{\bar{\tau }}_{\ell }$. Here, N is fixed and ϖ may depend on N; throughout this section, this bounded-propagation property is understood in the finite-N sense.*

**Theorem 3.** 
*Assume that the conditions of Theorem 1 and Corollary 1 hold, the delay bounds ${\bar{\tau }}_{\ell }$ and $\mu_{\ell }$ are finite, and the matrices $P_{s},Q_{\ell },R_{\ell }$ are uniformly positive definite and uniformly bounded over all modes. Then,*

*1.*  *If w(t)≡0, the closed-loop platoon has mean-square $L_{\infty }$ bounded propagation in the sense of Definition 2;*
*2.*  *If $∥w\left(t\right)∥\leq w_{\max}$, the local errors satisfy the following practical bounded-propagation estimate*
(40) $$\underset{t\to \infty }{\lim\sup}\underset{i}{\sup}E\{∥\delta_{i}\left(t\right){∥}^{2}\}\leq \gamma_{0}w_{\max}^{2}$$
  *for some $\gamma_{0}>0$ independent of i;*
*3.*  *If w(t)→0, then*
(41) $$\lim_{t\to \infty }\underset{i}{\sup}E\{∥\delta_{i}\left(t\right){∥}^{2}\}=0.$$

**Proof.**  Corollary 1 provides the tail ISS estimate

(42) $$\begin{array}{cc} E\{∥\zeta \left(t\right){∥}^{2}\}\leq & c_{1}e^{-\alpha (t-t_{0})}\underset{t_{0}-{\bar{\tau }}_{\max}\leq s\leq t_{0}}{\sup}{E\{∥\zeta \left(s\right)∥}^{2}\}\\ \; & +c_{2}\underset{t_{0}\leq s\leq t}{\sup}{∥w\left(s\right)∥}^{2} \end{array}$$

for all $0\leq t_{0}\leq t$ and for constants $c_{1},c_{2},\alpha >0$ that do not depend on the vehicle index *i*. Because $\delta_{i}$ is a subvector of ζ, we have

(43) $$\underset{i}{\sup}E\{∥\delta_{i}{\left(t\right)∥}^{2}{\}\leq E\{∥\zeta \left(t\right)∥}^{2}\}.$$

When w≡0, inequality (42) with $t_{0}=0$ reduces to

(44) $$\underset{i}{\sup}E\{∥\delta_{i}{\left(t\right)∥}^{2}\}\leq c_{1}e^{-\alpha t}\underset{-{\bar{\tau }}_{\max}\leq s\leq 0}{\sup}{E\{∥\zeta \left(s\right)∥}^{2}\}.$$

Using the fixed finite platoon size,

$${E\{∥\zeta \left(s\right)∥}^{2}\}\leq N\underset{i}{\sup}E\{∥\delta_{i}\left(s\right){∥}^{2}\}.$$

Choose

(45) $$\varpi^{2}<\frac{\epsilon^{2}}{c_{1}N},$$

then (39) follows from (44).

For bounded nonzero *w*, setting $t_{0}=0$ in (42) and letting t→∞ yields (40) with $\gamma_{0}=c_{2}$.

Finally, suppose that w(t)→0. Let η>0 be arbitrary. Choose $t_{0}$ sufficiently large that ${∥w\left(s\right)∥}^{2}\leq \eta$ for all $s\geq t_{0}$. Applying (42) with this $t_{0}$ gives

$$\underset{i}{\sup}E\{∥\delta_{i}{\left(t\right)∥}^{2}\}\leq c_{1}e^{-\alpha (t-t_{0})}\underset{t_{0}-{\bar{\tau }}_{\max}\leq s\leq t_{0}}{\sup}{E\{∥\zeta \left(s\right)∥}^{2}\}+c_{2}\eta .$$

Letting t→∞, the first term vanishes and

$$\underset{t\to \infty }{\lim\sup}\underset{i}{\sup}E\{∥\delta_{i}\left(t\right){∥}^{2}\}\leq c_{2}\eta ,$$

since η>0 is arbitrary, (41) follows. This completes the proof. □

**Remark 2.** 
*The result of Theorem 2 concerns bounded propagation of the local state-error vector in the mean-square $L_{\infty }$ sense. It recovers the bounded-propagation notion employed by Ding et al. [13] and extends it to heterogeneous link-dependent time-varying delays. It does not assert exact zero tracking during nonzero leader acceleration, which is consistent with the ISS scope of Corollary 1.*

## 4. Simulation Results and Discussion

### 4.1. Configuration of Experiment

**Figure 2:**
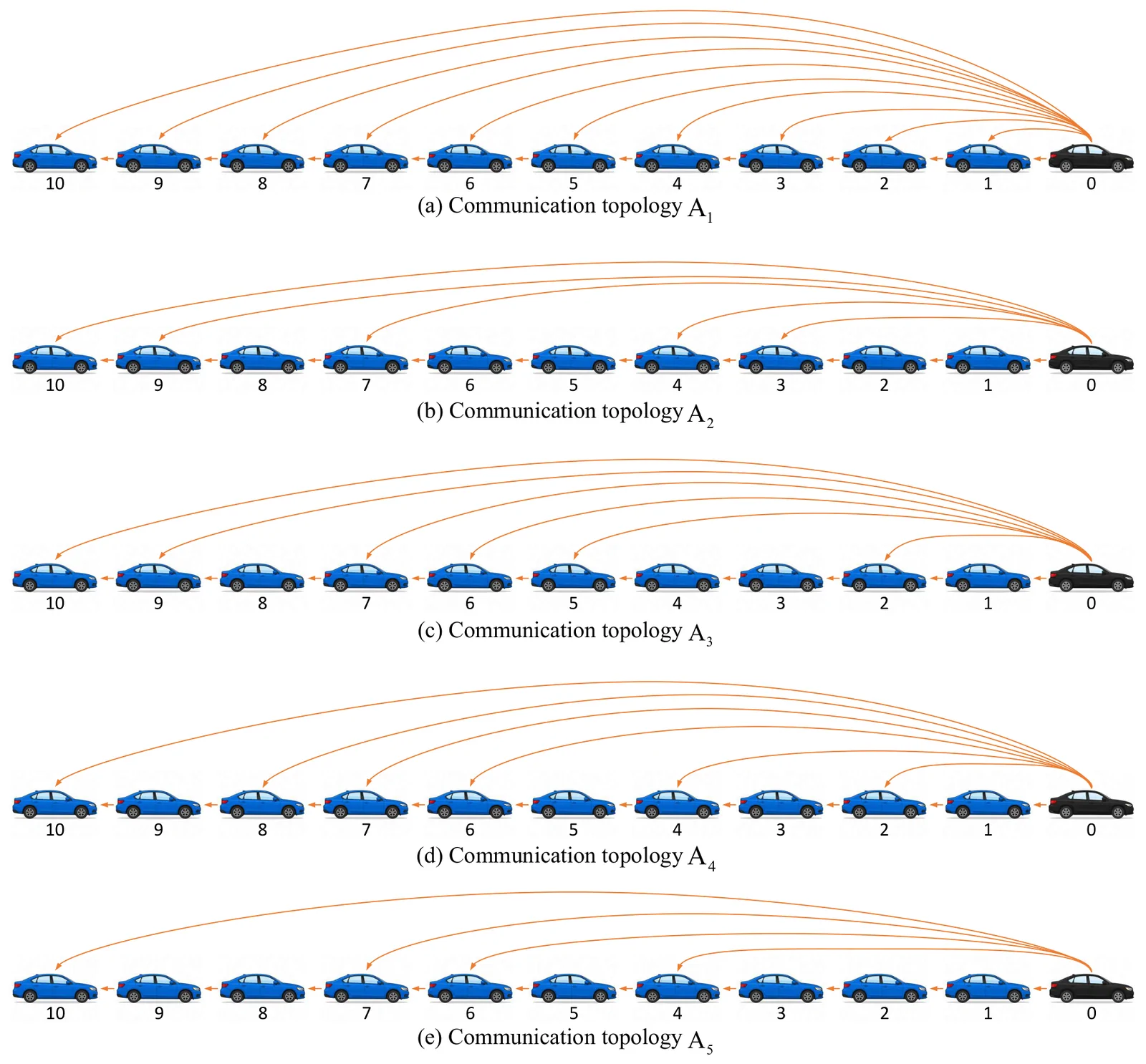
The proposed communication-topology structure [24].

A heterogeneous platoon with one leader and ten follower vehicles is simulated. Figure 2 shows the five communication topologies. Table 1 lists the parameter values, and the control gains are obtained by solving the associated LMI problem. For Theorem 1, all delay channels use ${\bar{\tau }}_{\ell }=0.10$ s and $\mu_{\ell }=0.04$. The resulting feasible solution confirms that the selected gains satisfy the nominal LMI condition under the prescribed delay assumptions.

Because LMI feasibility alone is insufficient to assess time-domain performance, we performed a controlled perturbation comparison using the same error model, controller gains, Markov mode sequence, and initial history. The leader acceleration was set to zero, and a 1 m initial position-error perturbation was applied to the first follower. This separates the effect of the delay-bound allocation from the time-varying leader excitation in the EUDC simulation. In the unified case, all nine follower-to-follower links were assigned the worst-case delay of 0.60 s. In the heterogeneous case, eight links were assigned 0.10 s and one link 0.60 s. The corresponding LMIs were infeasible and feasible, respectively. Table 2 summarizes the delay assignments and the resulting time-domain metrics. Over the full 450 s horizon, the heterogeneous allocation reduced the RMS position error from 0.01474 m to 0.01357 m, a reduction of 7.9%; reduced the RMS adjacent spacing error from 0.01280 m to 0.01127 m, a reduction of 12.0%; and shortened the time to settle below 0.01 m from 26.94 s to 19.81 s, a reduction of 26.5%. These results indicate that retaining link-specific delay bounds reduces the conservatism of assigning the worst-case delay bound to every communication link.

The communication topology switches among five modes according to the CTMC σ(t), whose transition-rate matrix is

(46) $$\Pi =\left[\begin{array}{ccccc} -0.060 & 0.015 & 0.015 & 0.015 & 0.015\\ 0.027 & -0.090 & 0.027 & 0.018 & 0.018\\ 0.015 & 0.0225 & -0.075 & 0.0225 & 0.015\\ 0.030 & 0.030 & 0.030 & -0.120 & 0.030\\ 0.012 & 0.012 & 0.012 & 0.024 & -0.060 \end{array}\right].$$

The off-diagonal rates characterize the switching intensity, and the EUDC provides the leader velocity trajectory.

### 4.2. Results and Discussion

**Figure 3:**
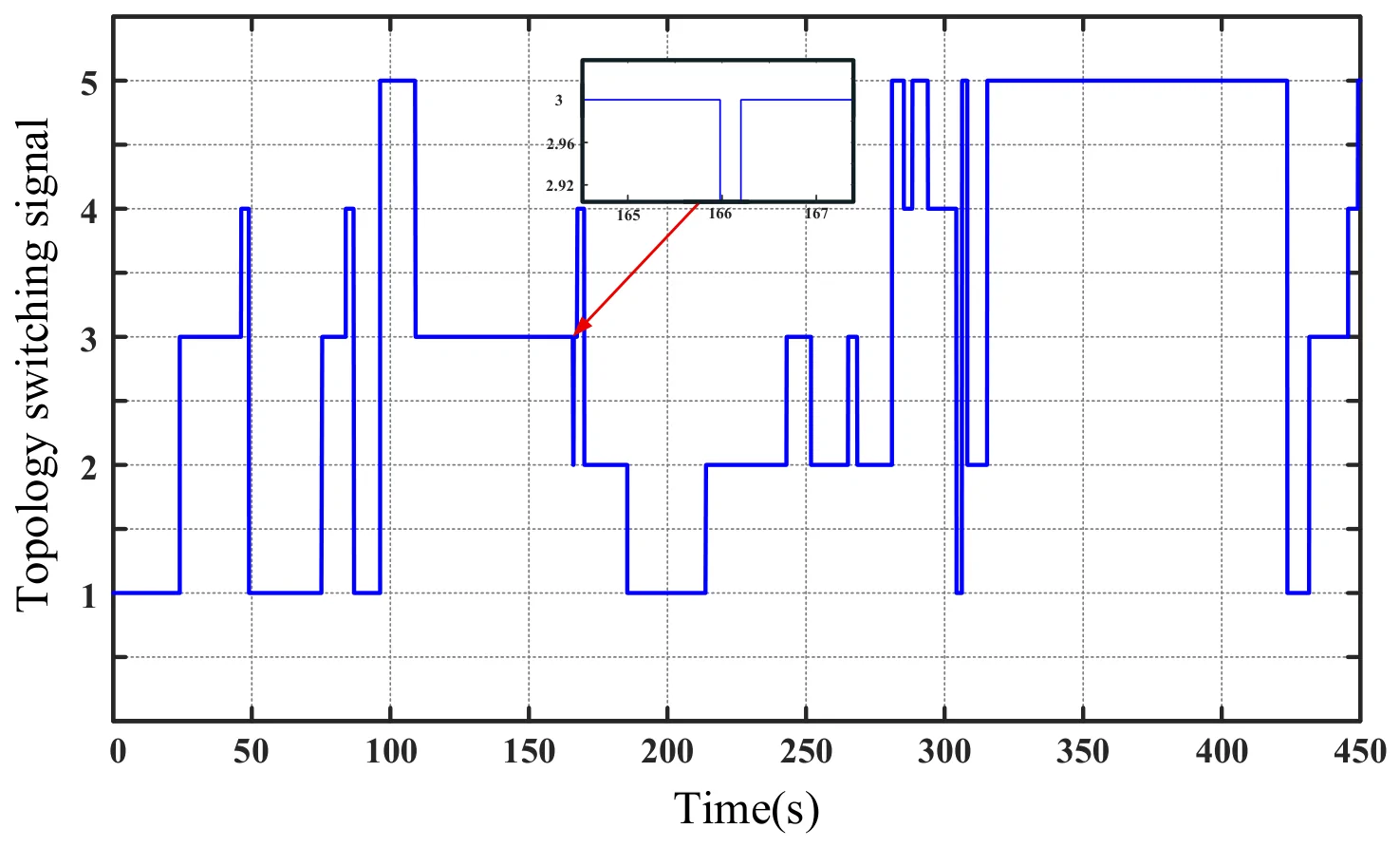
Markovian switching signal.

**Figure 4:**
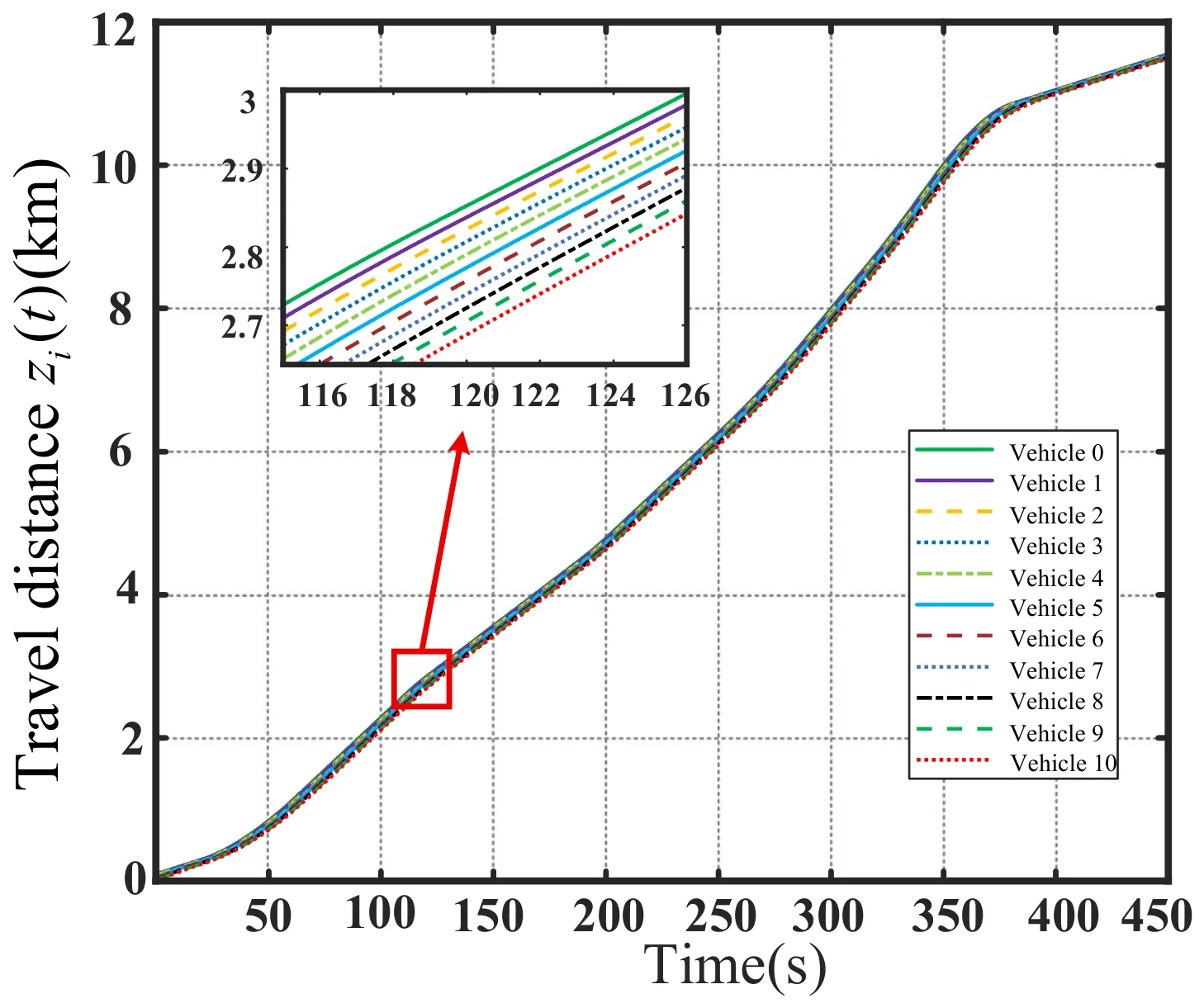
Position responses of the vehicles.

**Figure 5:**
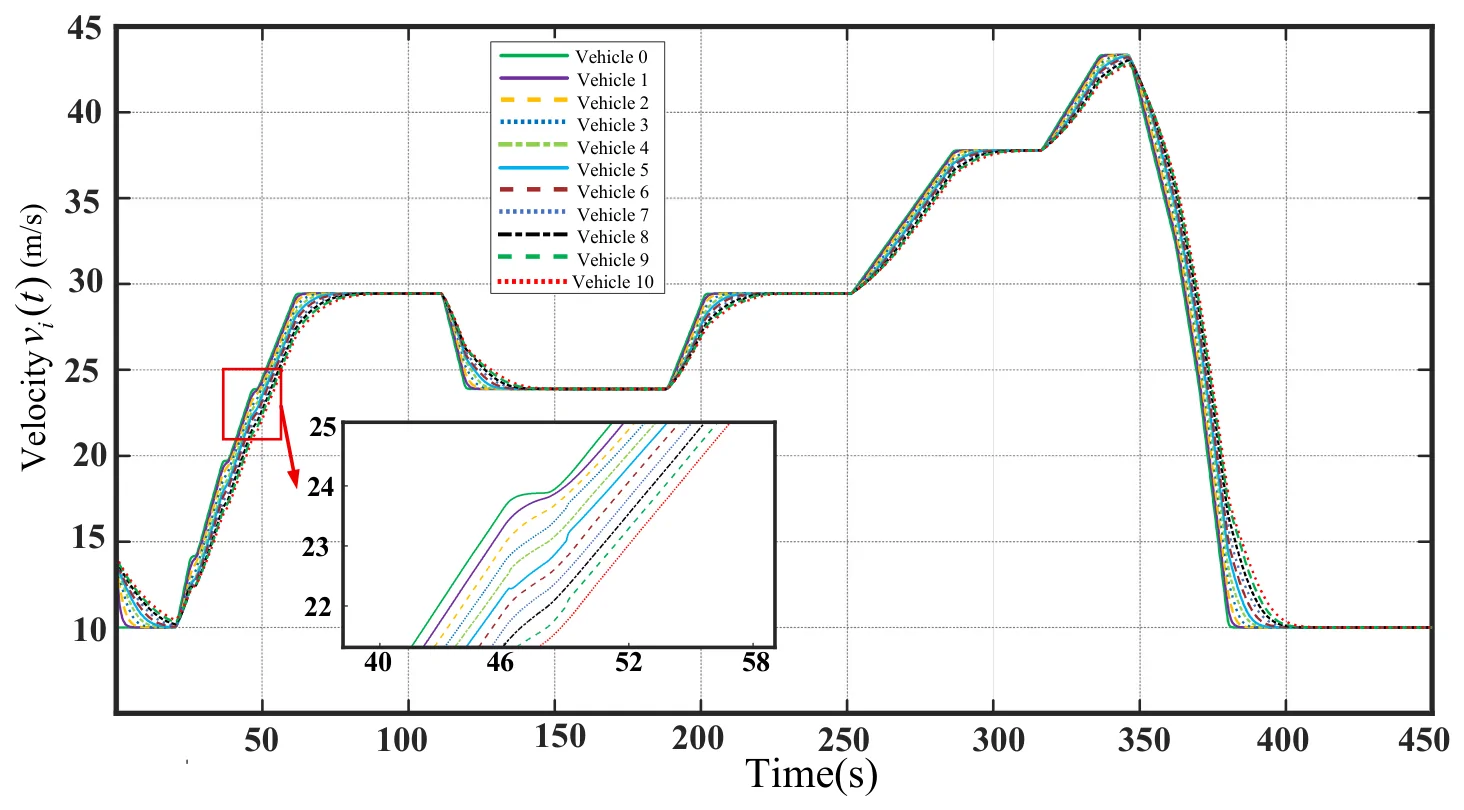
Velocity responses of the vehicles.

**Figure 6:**
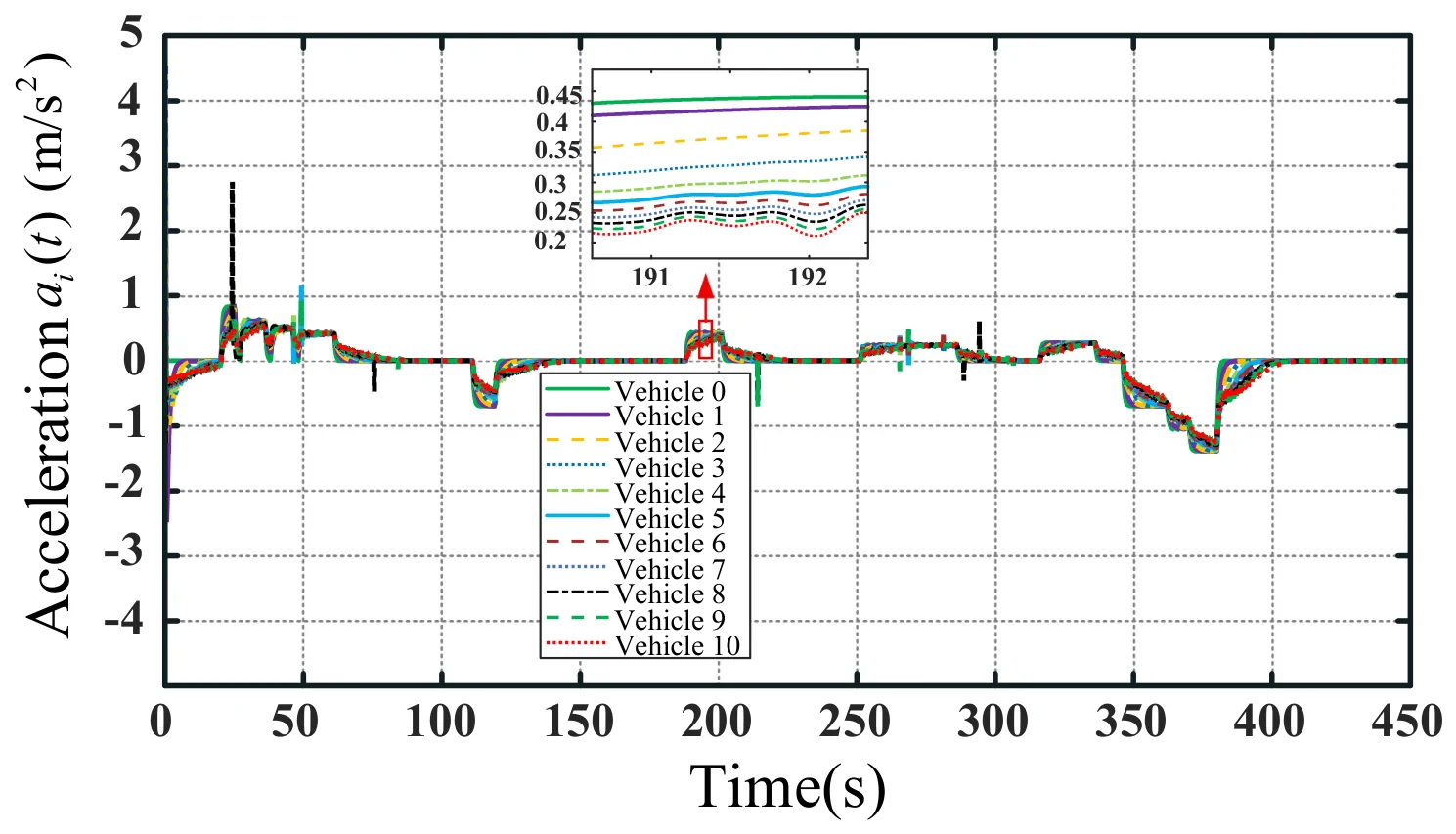
Acceleration responses of the vehicles.

Simulations were performed over t∈[0,450] s. Figure 3, Figure 4, Figure 5 and Figure 6 show the switching signal and the position, velocity, and acceleration responses. No position trajectories cross, velocities follow the leader profile, and transient tracking errors appear during strong acceleration and deceleration.

The early acceleration peak is 4.58 m/s^2^ at approximately 0.24 s, before the first topology transition at about 24 s, so this peak is not caused solely by switching. During 200–300 s, the leader acceleration magnitude remains below 0.45 m/s^2^, and the maximum follower acceleration decreases to 0.70 m/s^2^ despite nine transitions. During 400–450 s, the leader acceleration is effectively zero and all follower acceleration magnitudes remain below 0.10 m/s^2^ despite four further transitions. These trends indicate recovery toward coordinated motion.

Leader pinning provides the common reference, delayed neighbor coupling maintains local coordination, and the CTH/FVD feedback regulates spacing and velocity. Topology switching therefore changes the available information paths rather than directly determining the response magnitude.

**Figure 7:**
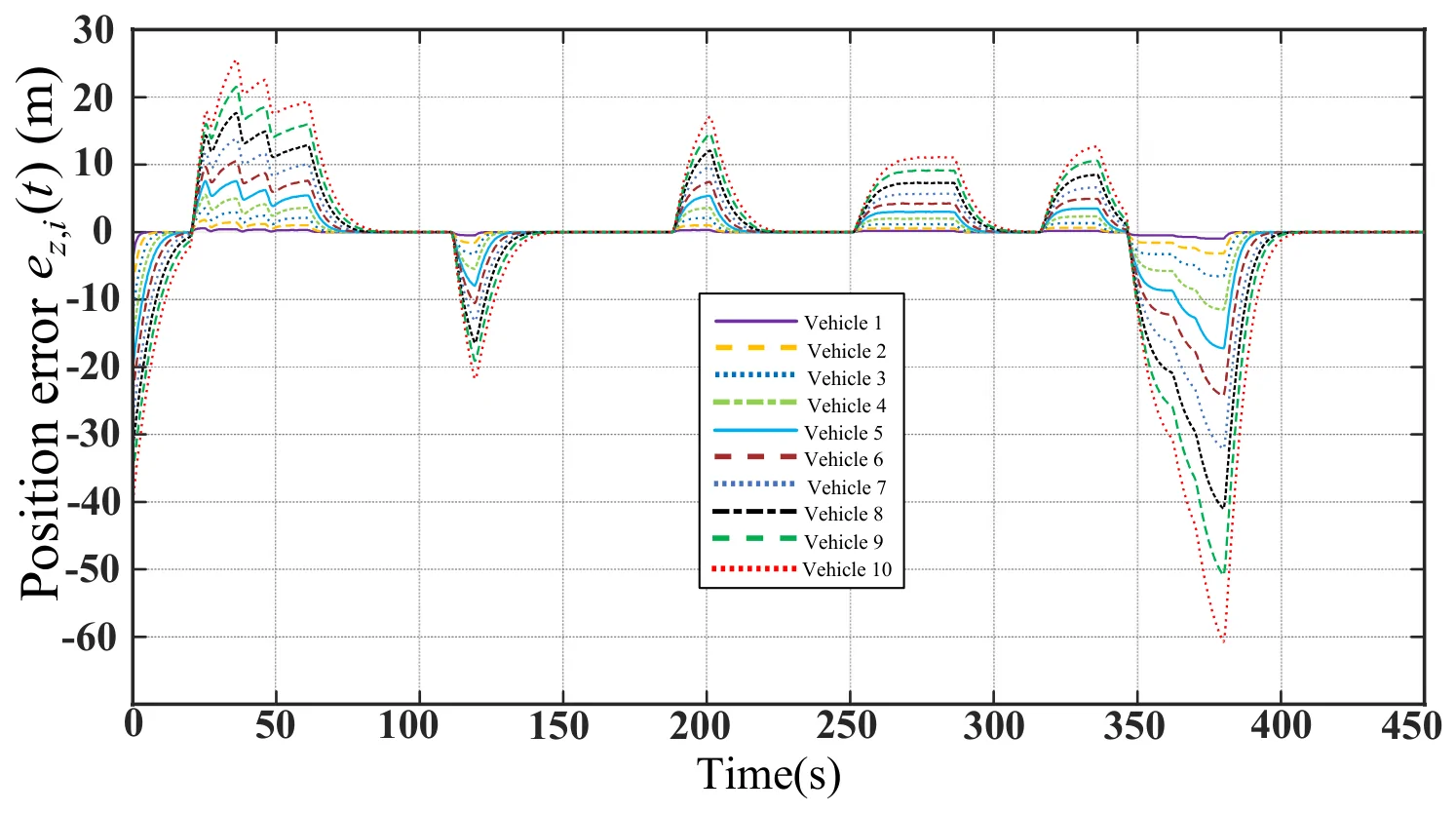
Position error responses of followers.

Figure 7 shows that $e_{z,10}\left(t\right)$ reaches −60.75 m at approximately t=380 s during strong braking. The leader decelerates at about −1.39 m/s^2^, with speed decreasing from 17.6 m/s at 375 s to 10.0 m/s at 382 s. The CTH target moves forward as speed decreases, but finite powertrain inertia and heterogeneous communication delays prevent instantaneous follower deceleration. Consequently, the velocity error grows from 0.83 m/s to 7.48 m/s, and the position error from −0.95 m to −60.75 m. Because $e_{z,i}\left(t\right)=z_{i}\left(t\right)-z_{0}\left(t\right)-d_{i0}\left(t\right)<0$, the actual leader-relative separation is larger than the desired CTH offset; the negative error indicates tracking lag, not gap compression.

This transient does not imply loss of car-following safety. At t≈380 s, the gap between vehicles 9 and 10 is approximately 16.2 m and $W_{10}=1.54>0$. The minimum gap, 5.87 m, occurs during start-up at t≈26 s, and the minimum safety margin after the initial 10 s is 0.84. The position error measures CTH tracking accuracy, whereas $W_{i}>0$ only indicates that the actual gap exceeds the critical braking distance. Thus, a positive margin does not certify a small tracking error, and a large transient error does not by itself imply collision. $|e_{z,10}\left(t\right)|$ falls below 1 m at approximately 401 s.

**Figure 8:**
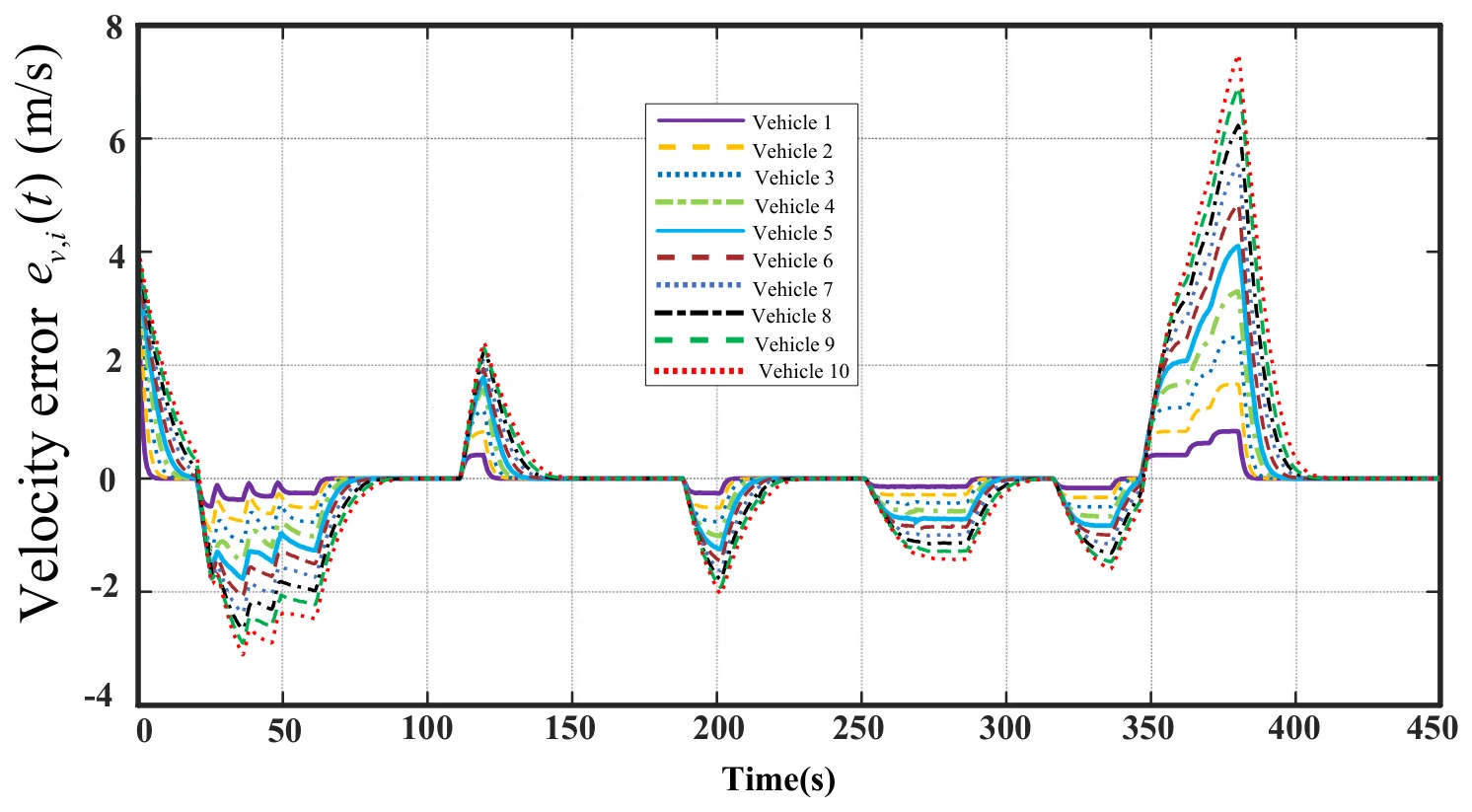
Velocity error responses of followers.

**Figure 9:**
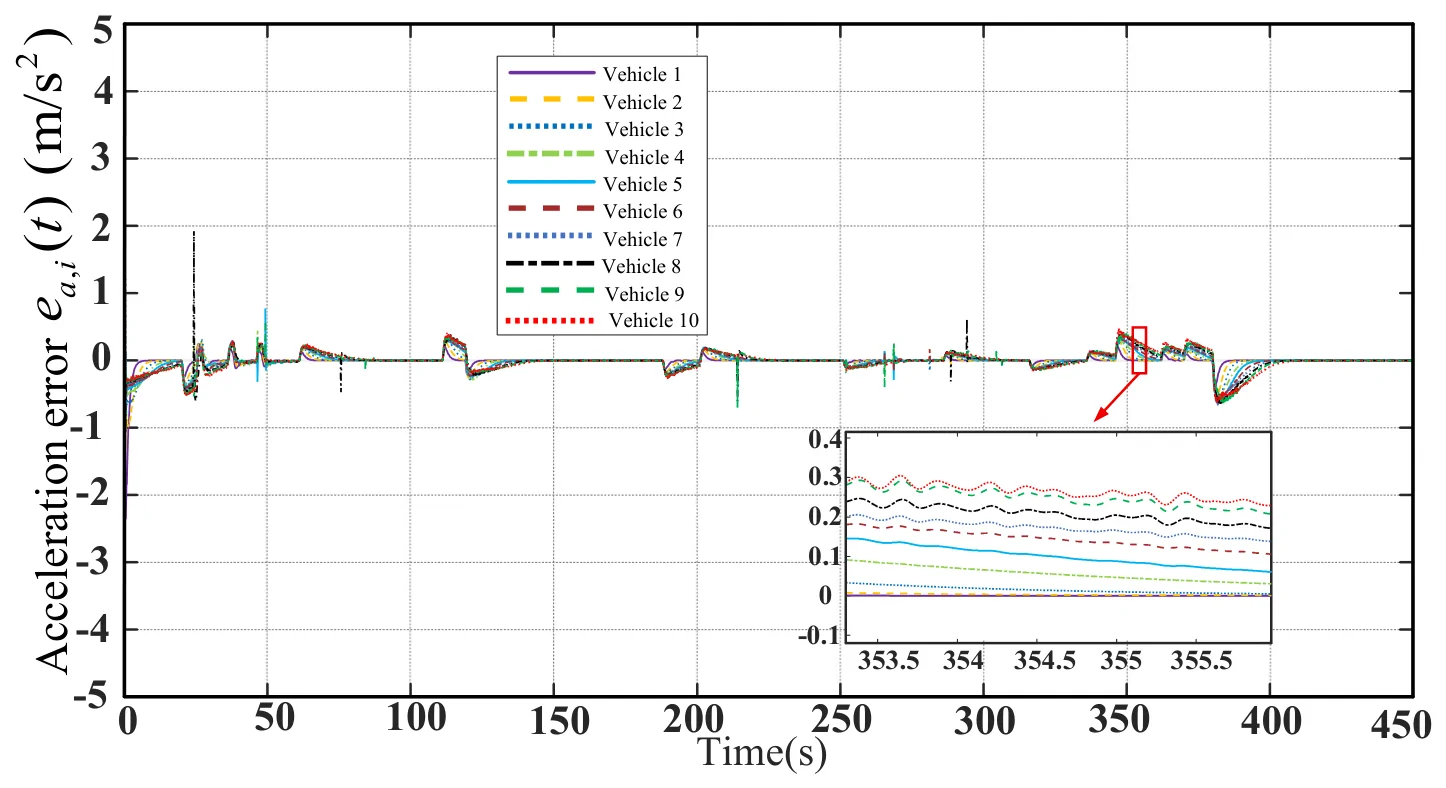
Acceleration error responses of followers.

The LMI certifies nominal mean-square stability under the stated delay assumptions, whereas the simulations evaluate the forced EUDC response. For the propagation assessment, the position-error peak increases from 0.95 m for Vehicle 1 to 60.75 m for Vehicle 10, and the maximum adjacent local peak ratio is 2.32; Figure 8 shows velocity-error peaks increasing from 0.83 to 7.48 m/s across the platoon. Figure 9 complements this assessment by showing the acceleration-error transients. Because the adjacent peak ratio exceeds 1, the present controller does not satisfy strict non-amplification string stability. The reported property is instead mean-square $L_{\infty }$ bounded propagation, following the bounded-propagation definition of Ding et al. [13]. A controller redesign with explicit local-error coupling and an $H_{\infty }$-type non-amplification constraint remains future work.

A dimensionless vehicle-following safety margin index, denoted by $W_{i}\left(t\right)$, is defined as

(47) $$W_{i}\left(t\right)=\frac{s_{i}\left(t\right)-s_{i}^{\mathrm{br}}\left(t\right)}{v_{i}\left(t\right)\tau_{h}},\;i=1,2,\ldots ,N,$$

where $\tau_{h}$ denotes the safety reaction-time parameter, and $s_{i}^{\mathrm{br}}\left(t\right)$ represents the critical braking distance. The expression for $s_{i}^{\mathrm{br}}\left(t\right)$ is given by

(48) $$s_{i}^{\mathrm{br}}\left(t\right)=\Delta v_{i}\left(t\right)\left(\xi_{i}+\tau_{s,i}\right)+\varphi_{r}\left[\frac{v_{i}^{2}\left(t\right)-v_{i-1}^{2}\left(t\right)}{2a_{\max}}\right].$$

Here, $s_{i}^{\mathrm{br}}\left(t\right)$ is the critical braking distance in (48). A positive $W_{i}\left(t\right)$ indicates a gap exceeding this distance, but not necessarily a small spacing error. Table 3 lists the parameters.

**Figure 10:**
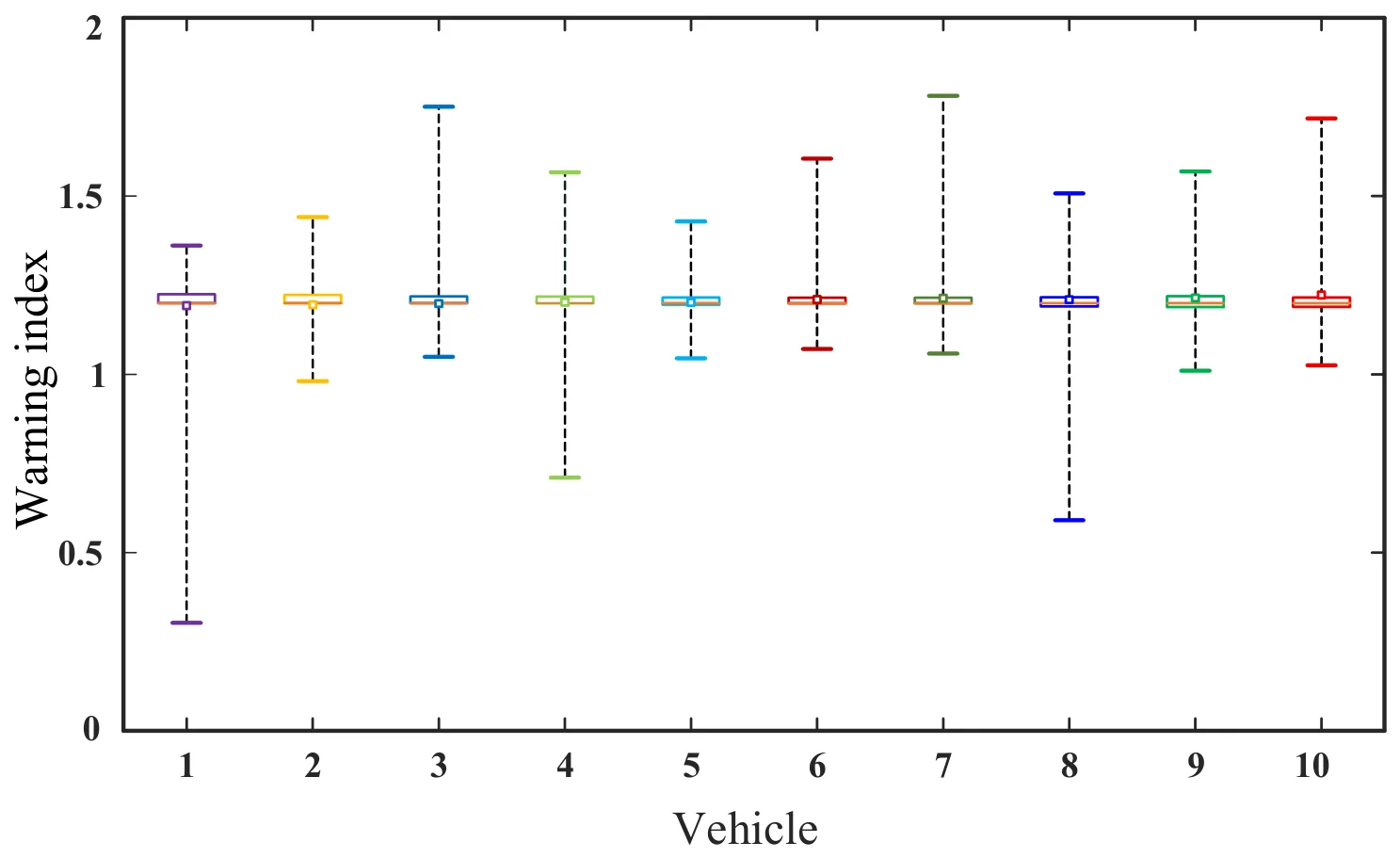
Safety margin of the follower vehicles under the EUDC.

Figure 10 shows that the safety-margin values are mostly concentrated around 1.2, with consistent mean and median values, indicating positive margins throughout most of the EUDC.

### 4.3. Sensitivity to Delay Bound and Switching Rate

Table 4 summarizes the robustness margins. For the delay-bound sweep, a coarse scan shows feasibility at 0.30 s and infeasibility at 0.375 s. The reported refined LMI checks include 0.3050 s as a feasible point and 0.3060 s as an infeasible point, giving the numerical feasibility bracket [0.3050,0.3060] s with a bracket width of 0.0010 s. Because the LMI is a sufficient condition and is solved with finite numerical tolerances, the bracket is interpreted as the numerical feasibility boundary of the tested LMI, not as the exact stability boundary of the underlying system. For the switching-rate study, the off-diagonal entries of Π are scaled by *c*, and the diagonal entries are recomputed so that every row sums to zero. This study is intentionally performed on a 12 s linearized Markov delay error model rather than on the full 450 s EUDC simulation. This experiment is designed to isolate the local effect of topology-switching rate, not to reproduce the complete driving-cycle response. The local perturbation is applied at t=3 s and lasts 2.4 s. At the fastest tested switching rate (c=8), four mode transitions occur within the 12 s horizon, with a mean dwell time of 2.4 s. Thus, the 12 s window covers the complete local perturbation and a representative sequence of rapid topology switches while avoiding confounding of the switching-rate effect with the long-duration EUDC excitation. The 450 s EUDC simulation remains the primary full-cycle validation and is not replaced by this local sensitivity analysis. As *c* increases from 0.5 to 8, the number of mode transitions increases and the mean dwell time decreases, whereas the maximum local position-error peak remains approximately 0.200–0.202 m. The LMI remains feasible at c=8.

### 4.4. Computational Complexity and Scalability

The LMI conditions of Theorem 1 are solved offline at the controller-design stage. Since $\zeta \left(t\right)\in R^{3N}$, the augmented state collects $1+2m_{d}$ copies of ζ(t), giving

(49) $$\dim\chi \left(t\right)=\left(1+2m_{d}\right)\dim\zeta \left(t\right)=3N\left(1+2m_{d}\right).$$

For each topology mode, the LMI consists of one block of dimension $3N(1+2m_{d})$ and $m_{d}$ blocks of dimension 3N, so its overall dimension is $3N(1+3m_{d})$. The number of scalar decision variables is $\left(q+2m_{d}\right)\;3N\left(3N+1\right)/2$.

For N=10, $m_{d}=9$, and q=5, the augmented state has dimension 570, each mode-dependent LMI has dimension 840, and the optimization involves 10,695 scalar decision variables. The problem was solved with MOSEK 11.1 called from YALMIP(R20250626) in MATLAB R2024b on an Intel Core i5-13500HX platform with 16 GB of memory. LMI assembly required about 2.6 s, and the interior-point solve required about 201 s after 39 iterations. This cost is incurred once offline; once the gains are fixed, the online control law depends only on local and neighboring states.

Table 5 reports sizes for N=5,10,20 with $m_{d}=N-1$, consistent with the union of the five topology modes. For fixed $m_{d}$, dimχ(t) and the LMI dimension grow linearly with *N*, while the number of decision variables grows quadratically. When $m_{d}$ grows with *N*, the augmented-state and LMI dimensions grow quadratically, and the decision-variable count grows cubically. The proposed synthesis is therefore tractable at the present scale but becomes limiting for very long platoons; sparse or decentralized synthesis is a natural future direction.

## 5. Conclusions and Future Directions

This paper studies distributed cooperative control of connected vehicle platoons subject to Markovian switching communication topologies and link-dependent heterogeneous time-varying delays. A closed-loop error model and distributed control protocol incorporating powertrain inertial lag, leader-pinning information, and local inter-follower communication coupling are developed. Based on a mode-dependent Lyapunov–Krasovskii functional, Jensen’s integral inequality, and the Schur complement, sufficient LMI conditions are derived for the mean-square asymptotic stability of the nominal closed-loop system under the prescribed continuously differentiable time-varying delay conditions. An ISS corollary extends the analysis to bounded time-varying leader acceleration and establishes bounded tracking during dynamic reference phases, with convergence when the leader acceleration and its derivative vanish. Numerical simulations under the Extra-Urban Driving Cycle (EUDC) show that, despite Markovian topology switching and bounded communication delays, the position, velocity, and acceleration errors of the follower vehicles remain bounded during the dynamic portions of the cycle and converge as the leader acceleration vanishes. Meanwhile, the dimensionless car-following safety-margin indices remain positive throughout the simulation, demonstrating coordinated tracking and safe car-following under the considered operating conditions.

The present work establishes, for a fixed finite platoon size *N*, a mean-square $L_{\infty }$ bounded-propagation property in the sense of Ding et al. [13]. This property does not imply strict frequency-domain or non-amplification string stability, which is beyond the scope of the present study and will be investigated in future work. The current framework does not explicitly address road-gradient variations, aerodynamic drag, or model parameter uncertainty. Future work will extend it to packet loss, intermittent communication, and disturbance estimation and compensation for real-world driving.

## Figures and Tables

**Table 1:** Simulation parameters.

Parameters and Notations	Symbol	Value
Powertrain inertial-lag time constant	$\xi_{i}$	$\xi_{0}=0.45\;s\;\left[\xi_{1},\ldots ,\xi_{10}\right]={\left[0.35,0.42,0.32,0.53,0.58,0.47,0.42,0.53,0.58,0.47\right]}^{T}s$
Time headway	*h*	0.6
FVD linearization coefficients	$\phi_{ij}$	$\phi_{ij}\in \left[-0.7356,\;0.7356\right]$
FVD adjustment parameter	δ	−32.60
Velocity-channel adjustment parameter	μ	−6.85
Local error-feedback gain	$\gamma_{i}$	$\left[\gamma_{1},\ldots ,\gamma_{10}\right]={\left[50.60,\;51.80,\;50.90,\;52.40,\;51.50,50.20,\;52.10,\;51.00,\;52.60,\;50.80\right]}^{T}$
Velocity–acceleration channel weight	$\rho_{i}$	$\left[\rho_{1},\ldots ,\rho_{10}\right]={\left[575,\;592,\;580,\;600,\;588,570,\;595,\;582,\;605,\;578\right]}^{T}$
Leader pinning edge weights	$k_{i0}$	[$k_{1,0},\ldots ,k_{10,0}$] = [10,450, 12,150, 12,500, 12,280, 12,620, 12,080, 12,430, 12,300, 12,700, 12,220]^*T*^
Adjacent following edge weights	$k_{i,i-1}$	$\left[k_{2,1},\ldots ,k_{10,9}\right]={\left[7763.8,\;7966.3,\;7669.2,\;8101.3,7804.3,\;7993.3,\;7709.7,\;8155.3,\;7898.8\right]}^{T}$
Communication delay range	$\tau_{\ell }\left(t\right)$	$0\leq \tau_{\ell }\left(t\right)\leq 0.10\;s$

**Table 2:** Controlled 450 s delay-allocation comparison under a fixed controller and the nine-channel error model. RMS metrics are computed over all followers and the full 450 s horizon; settling time is the first time after which $\max_{i}\left|e_{z,i}\left(t\right)\right|$ remains below 0.01 m.

Metric	Unified Bound	Heterogeneous Bounds
Link delay assignment	All 9 links: 0.60s	8 links: 0.10s; 1 link: 0.60s
LMI feasibility	No	Yes
RMS position error (m)	0.01474	0.01357
RMS adjacent spacing error (m)	0.01280	0.01127
Settling time to 0.01m (s)	26.94	19.81

**Table 3:** Parameters of the Dimensionless Safety Margin Index.

Parameter	Symbol	Value
Safety reaction time	$\tau_{h}$	0.5s
Equivalent response delay of the safety index	$\tau_{s,i}$	$0\leq \tau_{s,i}\leq 0.10\;s$
Risk correction coefficient	$\varphi_{r}$	1.4
Maximum braking deceleration magnitude	$a_{\max}$	$8\;m/s^{2}$

**Table 4:** Sensitivity to delay bound and topology switching rate.

**Panel A: delay bound**								
Delay bound ${\bar{\tau }}_{\ell }$ (s)	0.1000	0.2500	0.3000	0.3050	0.3060	0.3100	0.3750	0.5000
LMI feasible	Yes	Yes	Yes	Yes	No	No	No	No
**Panel B: switching rate (12 s linearized model)**								
Scale *c*	0.5	1	2	4	8			
Switches in 12 s	0	0	2	3	4			
Mean dwell (s)	12.0	12.0	4.0	3.0	2.4			
Max local position peak (m)	0.2000	0.2000	0.2000	0.2020	0.2000			
LMI at c=8	Feasible							

**Table 5:** Problem size of the LMI conditions in Theorem 1 for representative platoon lengths (q=5 modes, $m_{d}=N-1$ delay channels).

*N*	$m_{d}$	dimχ(t)	LMI Dimension	Scalar Decision Variables
5	4	135	195	1560
10	9	570	840	10,695
20	19	2340	3480	78,690

## Data Availability

The data generated during the current study are available from the corresponding author upon reasonable request.

## Nomenclature

The following list summarizes the principal state variables and analytical quantities. Parameters reported in the simulation tables are not repeated here. Auxiliary matrices and constants are defined where they are introduced.

**Symbol**	**Description**
$z_{i}\left(t\right),v_{i}\left(t\right),a_{i}\left(t\right)$	Longitudinal position, velocity, and acceleration of vehicle *i*
$u_{i}\left(t\right)$	Control input of follower *i*
$d_{i0}\left(t\right)$	Desired position offset of follower *i* relative to the leader
$e_{z,i}\left(t\right),e_{v,i}\left(t\right),e_{a,i}\left(t\right)$	Leader-relative position, velocity, and acceleration tracking errors
ζ(t)	Stacked position, velocity, and acceleration error vector
$\delta_{i}\left(t\right)$	Local state-error vector of follower *i*
$q_{i}\left(t\right)$	Difference between the position-tracking errors of adjacent vehicles
σ(t)	Active communication-topology mode
$\Pi =[\pi_{sr}]$	Markov transition-rate matrix
$\alpha_{ij}^{\left(s\right)},\alpha_{i0}^{\left(s\right)}$	Inter-follower connectivity and leader-pinning indicators in mode *s*
$\kappa_{ij}^{\left(s\right)},\kappa_{i0}^{\left(s\right)}$	Effective inter-follower coupling and leader-pinning weights
$c_{ij}^{\left(s\right)},c_{i0}^{\left(s\right)}$	Equivalent position-coupling coefficients
$\mu_{\ell }$	Upper bound on the rate of change of the delay on link *ℓ*
$P_{s},Q_{\ell },R_{\ell }$	Symmetric positive-definite matrices in the Lyapunov–Krasovskii functional
w(t)	Exogenous input comprising the leader acceleration and its derivative
$W_{i}\left(t\right)$	Dimensionless car-following safety margin
$s_{i}\left(t\right),s_{i}^{\mathrm{br}}\left(t\right)$	Actual inter-vehicle distance and critical braking distance
